# Microbiome dynamics during the HI-SEAS IV mission, and implications for future crewed missions beyond Earth

**DOI:** 10.1186/s40168-020-00959-x

**Published:** 2021-01-24

**Authors:** Alexander Mahnert, Cyprien Verseux, Petra Schwendner, Kaisa Koskinen, Christina Kumpitsch, Marcus Blohs, Lisa Wink, Daniela Brunner, Theodora Goessler, Daniela Billi, Christine Moissl-Eichinger

**Affiliations:** 1 Interactive Microbiome Research, Diagnostic & Research Institute of Hygiene, Microbiology and Environmental Medicine, Medical University of Graz, Neue Stiftingtalstrasse 6, 8010 Graz, Austria; 2 Laboratory of Applied Space Microbiology, Center of Applied Space Technology and Microgravity (ZARM), University of Bremen, Am Fallturm 2, 28359 Bremen, Germany; 3 University of Florida, Space Life Sciences Lab, 505 Odyssey Way, Exploration Park, N. Merritt Island, FL 32953 USA; 4 BioTechMed-Graz, Graz, Austria; 5 Department of Biology, University of Rome Tor Vergata, Via della Ricerca Scientifica s.n.c, 00133 Rome, Italy

**Keywords:** Confined built environments, Isolation, HI-SEAS, Skin microbiome, Indoor microbiome, 16S rRNA gene amplicons, qPCR, Antimicrobial resistances, Longitudinal, Phenotype predictions

## Abstract

**Background:**

Human health is closely interconnected with its microbiome. Resilient microbiomes in, on, and around the human body will be key for safe and successful long-term space travel. However, longitudinal dynamics of microbiomes inside confined built environments are still poorly understood. Herein, we used the Hawaii Space Exploration Analog and Simulation IV (HI-SEAS IV) mission, a 1 year-long isolation study, to investigate microbial transfer between crew and habitat, in order to understand adverse developments which may occur in a future outpost on the Moon or Mars.

**Results:**

Longitudinal 16S rRNA gene profiles, as well as quantitative observations, revealed significant differences in microbial diversity, abundance, and composition between samples of the built environment and its crew. The microbiome composition and diversity associated with abiotic surfaces was found to be rather stable, whereas the microbial skin profiles of individual crew members were highly dynamic, resulting in an increased microbiome diversity at the end of the isolation period. The skin microbiome dynamics were especially pronounced by a regular transfer of the indicator species *Methanobrevibacter* between crew members within the first 200 days. Quantitative information was used to track the propagation of antimicrobial resistance in the habitat. Together with functional and phenotypic predictions, quantitative and qualitative data supported the observation of a delayed longitudinal microbial homogenization between crew and habitat surfaces which was mainly caused by a malfunctioning sanitary facility.

**Conclusions:**

This study highlights main routes of microbial transfer, interaction of the crew, and origins of microbial dynamics in an isolated environment. We identify key targets of microbial monitoring, and emphasize the need for defined baselines of microbiome diversity and abundance on surfaces and crew skin. Targeted manipulation to counteract adverse developments of the microbiome could be a highly important strategy to ensure safety during future space endeavors.

Video abstract

**Supplementary Information:**

The online version contains supplementary material available at 10.1186/s40168-020-00959-x.

## Background

This decade may see the beginning of a sustainable human presence on the Moon. The US government stated their commitment to lead such an endeavor in the Space Policy Directive-1 [1] and current goals include landing a crew at the Moon’s south pole by 2024, before establishing a sustained presence there by 2028 [2]. Since 2015, the European Space Agency (ESA) has been strongly advocating its “Moon Village” concept, a large collaborative undertaking that would lead to a permanent presence on the Moon [3]. In addition, the latest Council at ministerial level anticipates a strong involvement of ESA in the US-led Moon program [4]. Other collaborators include the Japan Aerospace Exploration Agency (JAXA) and the Canadian Space Agency (CSA). While lunar exploration could greatly benefit different areas of science and technology in itself [5, 6], the Moon is expected to serve as a testing ground for crewed missions to Mars. Reaching the red planet is also the stated goal of private spacecraft companies, notably SpaceX [7] who aims for a landing as early as in the 2020s.

In such endeavors, microorganisms will inevitably co-travel with the crew: they are thought to outnumber human cells in our bodies [8] and each individual releases millions of them every hour [9]. A microbe-free crewed mission is unrealistic, unethical, and undesirable, as our microbiome is essential to our health [10]. Microbial communities may however pose, if inadequately managed, serious threats to future missions.

The most obvious threats to the crew’s health are pathogens. This risk is exacerbated by crew members’ confinement and proximity, limited treatment options, increased microbial transmission in microgravity [11], restricted hygienic practices, potentially increased virulence and decreased antibiotic susceptibility of bacteria in space [11–14], and lowered immune responses of astronauts attributed to microgravity, radiation, and stress [11, 12, 15–17]. Moreover, the lack of environmental microorganisms competing with human-borne pathogens for the same niche could complicate the establishment of resilient microbiomes in their habitat [18]. While no life-threatening infections have been reported during spaceflight so far, opportunistic pathogens, which are part of the normal human-associated microbial diversity, were detected on the International Space Station (ISS) [13, 19]. Such pathogens might have caused tens of minor medical incidents—among which are urinary tract, upper respiratory tract and subcutaneous skin infections—beyond Earth [11, 16].

The threat increases when emergency returns become impossible, notably on a Mars journey, which is expected to last at least 520 days [20]. Even in a relatively mild disease scenario, the resulting decrease in productivity may be a significant loss given the high value of astronaut working time.

Another threat comes from microorganisms’ interference with equipment. First, some microorganisms, referred to as technophiles, can colonize industrial materials and lead to hardware malfunction, degradation of structural materials, and corrosion of metal parts [21]. Technophiles were found onboard the Mir station [22, 23] and onboard the ISS [13, 19, 24], leading to damage of various systems [25, 26]. Second, microbial contaminants could interfere with biological life-support systems [27]. These life-support systems could greatly contribute to the feasibility of long-term missions on the Moon or Mars [28–30]. However, microbial contaminants could harm the system-relevant organisms through competition and/or toxicity or by making food products unsuitable for crew consumption.

Finally, microorganisms brought alongside the crew could interfere with the search for life on Mars [31, 32]. One strategy to mitigate the risk of contamination could be to increase our knowledge of, and to catalogue, the microbial communities we are carrying [33, 34]: this would help to discriminate between endogenous life and our microbiome in case of an ambiguous discovery and to assess the risk of contaminants to adapt to some local (micro)environments. While Mars’s surface appears hostile even to microbial life, it cannot be excluded that some extremophiles may reach niches where they could remain active [32].

In any case, microbial communities will be critical components of future space endeavors, with a potentially large influence on mission success [35]. Their importance is reflected in space agencies’ efforts to characterize them onboard the ISS [13, 19, 24, 36–40].

However, microbial monitoring on the ISS is constrained by logistical and funding challenges. An alternative is to study microbiome nature and dynamics in similar, closed systems (such as submarines and polar stations) or specific, ground-based analogues of long-term crewed spaceflight [18, 41]. While those analogues differ in some important aspects (e.g., gravity and radiation) from spaceflight itself, they place a small, isolated crew in combinations of the following: long-term confinement, high workloads, restricted waste disposal, limited hygiene, and/or low air or water quality. They offer the possibility to comprehensively monitor related medical and psychological issues.

In the past, several studies on the indoor and human microbiome were conducted in settings simulating missions to future facilities on the Moon or Mars, for instance: Mars500 (520 days) [41, 42], the Antarctic base Concordia (1 year) [43], the inflatable lunar/Mars analogous habitat (ILMAH) (30 days) [44], and the biological life-support testbed “Lunar Palace 1” (LP1; 105 days) [27]. For all these studies, individual parameters have to be critically considered such as variations in methodologies, environmental conditions, activity, geographical location, architectural design, baseline-diversity of microorganisms, contaminants from crew members, and cargo such as food and scientific equipment.

Another opportunity arose in 2015–2016: as part of the Hawaii Space Exploration Analog and Simulation IV (HI-SEAS IV) mission, six people spent 1 year in isolation in a dome (diameter: 11 m) located at 2.5 km of altitude on the barren slopes of the Mauna Loa volcano, primarily for NASA Behavioral Health and Performance (BHP) research [45]. Over a period of 336 days, swab and wipe samples from habitat surfaces and crew skin were taken in order to assess the microbial community fluctuation, as well as the interactions of surface and skin microbiomes.

Our main hypotheses were that (i) the microbiome of the HI-SEAS habitat would follow a longitudinal homogenization between individual crew members, but also the surrounding built environment; (ii) overall microbial diversity would be depleted; and (iii) the microbiome would resemble those of other long time experiments in isolated and confined built environments (ICE) on Earth and on the ISS.

## Methods

### Setting of the HI-SEAS IV mission

The 1-year Hawaii Space Exploration Analog and Simulation IV (HI-SEAS IV) mission took place from August 28^th^, 2015 to August 28^th^, 2016 [45] in the HI-SEAS habitat, an 11 m in diameter spherical-shaped dome located at 2.5 km of altitude on the barren slopes of the Mauna Loa volcano (Supplementary Fig. S1). Operated by the University of Hawaii, and funded by NASA, this habitat served primarily for NASA Behavioral Health and Performance (BHP) research. During HI-SEAS IV, six crew members (3 males and 3 females) selected for their astronaut-like profile, four from the USA and two from Europe (France and Germany), spent a year there in conditions mimicking those of a Mars mission. Time was mostly spent on research work (including outside work, wearing mock spacesuits), test subject duties, physical exercise, and household chores. The crew was physically isolated from other human beings for the whole mission duration and communications had a high latency (20-min delay in both directions). The diet was mostly composed of dehydrated food, canned food, occasional fermented food (yoghurt, bread, and cream cheese) made from rehydrated products, and rare vegetables grown on site.

Participants typically showered 1 to 3 times a week, for an average duration of 1.5 to 2 minutes under running water (Heinicke et al. submitted); bodies were then washed with Equate’s Sensitive Skin Body Wash, and hair with Garnier Fructis’ Pure Clean Clear 2in1. In-between showers, participants occasionally used disinfecting wipes (mainly, Kirkland’s Extra Large Disinfecting Wipes). Hands were routinely washed with Dr. Bronner’s 18-in-1 Hemp Peppermint Pure-Castile liquid soap, and disinfected with germ’s hand sanitizer.

A general cleaning of the habitat was performed every Sunday. Most hard surfaces were then cleaned with Simple Green’s cleaner, the kitchen floor with Comet’s bleach-based powder, and floors aside from the bathroom and kitchen were only vacuumed. Dishwashing was performed by hand. Clothes were washed with Kirkland’s UltraClean laundry detergent, either by hand or in a washing machine.

Sources of voluntarily introduced microorganisms included the following: Fermented products (sourdough bread, tempeh, cream cheese, kombucha, and yoghurt) were prepared using commercial microbial mixes. Toilets were composting toilets, maintained with Sun-Mar’s Microbe Mix and Sun-Mar’s compost swift. Cyanobacteria (*Anabaena* sp. PCC 7120 and *Chroococcidiopsis* sp. CCMEE 029) were used for research purposes. Part of the kitchen waste was processed in a bokashi composting system (purchased from Each One Teach One Farms, Hawaii).

### Sampling

Microbiome samples were taken every other week. Habitat/furniture surface samples were taken with swabs at  four different locations. Skin surface (front torso) samples were taken with wipes from each crew member. In-mission sampling occurred from September 4^th^, 2015 to August 5^th^, 2016, and an extra series of skin wipe samples was performed after mission completion. The baseline (day 0) was defined as the day of the first sampling event.

The swab samples were taken from (Supplementary Fig. S2):

- The front part of the (composting) toilet bowl (high density plastic), in the upstairs bathroom
- The kitchen floor (painted, waterproof plywood), in an area (between the fridge and another piece of furniture) where dust tended to accumulate
- The desk (medium density fiberboard overlaid with plastic laminate) in one of the bedrooms, and
- One desk (medium density fiberboard overlaid with plastic laminate) in the main room

For each habitat/furniture surface sample, a swab (552C regular swab; ethylene oxide sterilized, Copan, Brescia, Italy) was moistened with autoclaved, deionized water (ELGA’s Vision 125 Deionizer). An area of 5 × 5 cm was sampled in three directions (horizontal, vertical, and diagonal). The swab was turned between each change in direction. The swab was then broken at the predetermined breaking point and placed back into its original container. Field controls were performed during each sampling session by waving the swab in the air for a few seconds instead of sampling a surface.

Prior to samplings of skin surfaces, sterile 50-ml tubes were filled with one wipe (TX3211, SterileWipe LP, Texwipe) and 10 ml of autoclaved, deionized water. Once the wipes were homogeneously moistened, crew members sampled their own skin following oral and written instructions. Briefly, they put on gloves, cleaned them with ethanol, took the wipe out of the tube, put it flat on their hand, wiped their torso up and down and left and right, folded the wipe over the target surface, wiped again with one of the clean sides, wiped with the other side, and put the wipe back into the original tube. Twenty milliliters of water were then added to each tube before storage. Field controls were performed during each sampling session by waving the wipe in the air for a few seconds instead of wiping skin.

Swab and wipe samples were stored at − 20 °C after sampling, shipped to Europe in dry ice after mission completion and then stored at − 80 °C until analysis.

### DNA extraction

A total of 63 wipes were thawed at 4 °C overnight before transferring them to DNA-free bottles (baked at 250 °C for 24 h) filled with polymerase chain reaction (PCR)-grade water. The bottles were sonicated for 120 ± 5 s with a maximal power of 240 W and a frequency of 40 kHz, and vortexed at maximum speed for 1 min. The biomass-containing water suspension was concentrated to 200–500 μl using UV sterilized Amicon filters (Amicon Ultra 15 ml, 50 K, Merck Millipore).

DNA was then extracted from cells suspended and concentrated from wipes, and from all surface swabs plus 7 swab field controls (for a total of 111 swab samples), using QIAGEN’s DNeasy PowerSoil Kit.

### 16S rRNA gene amplicons

Microbial profiles were based on amplicons targeting the V4 region of the 16S rRNA gene. The common primer pair F515-R806 [46] with tags for Illumina sequencing was used to cover most bacterial and some archaeal taxa (Supplementary Table S1). Twenty-five microliters of the PCR reaction mix contained a final concentration of 200 mM each of forward and reverse primer (0.4 μl of 10 μM stock each), 0.1 μl TaKaRa ExTaq polymerase (5 U/μl, Clontech, Japan), 2 μl ExTaq buffer with MgCl_2_ (10×), 1.6 μl dNTP mix (2.5 mM), 2 μl of template DNA, and 18.5 μl PCR grade water. PCR conditions were as follows: initial denaturation at 94 °C for 3 min, followed by 35 cycles of denaturation at 94 °C for 45 s, annealing at 50 °C for 60 s, elongation at 72 °C for 90 s, and a final elongation step at 72 °C for 10 min. PCR fragments were evaluated for product size and quantity by agarose gel (3%) electrophoresis at 70 V for 30 min. Libraries for Illumina MiSeq sequencing were prepared by the Core Facility Molecular Biology at the Center for Medical Research at the Medical University Graz, Austria and covered biological samples, field blanks, extraction blanks, as well as no-template controls of PCRs. For the NGS library, DNA concentrations of the generated amplicons were normalized with a SequalPrep™ normalization plate (Invitrogen). After normalization of PCR products, each sample was indexed with a unique barcode sequence using 8 cycles of indexing PCR. Indexed samples were then pooled and purified by gel cuts. Finally, the library was sequenced on an Illumina MiSeq instrument and the MiSeq Reagent Kit v3, 602 cycles (2 × 301 cycles).

### Statistics and bioinformatics

After sequencing, resulting fastq files were processed with Qiime2 versions 2018.6–2020.8 [47]. Demultiplexed reads were denoised with DADA2 [48] and an amplicon sequence variant (ASV) feature table was created after truncating forward reads at position 200 and reverse reads at position 150. Potential contaminants were identified and removed with decontam [49] and its prevalence method with default settings (method = “prevalence,” neg=“is.neg,” threshold = 0.5). Representative sequences were classified by a Naïve Bayes trained classifier [50] based on Silva 128 [51, 52] and a rooted phylogenetic tree for phylogenetic diversity measures was created with Fasttree [53]. Core metrics for alpha and beta diversity (including metrics for richness, evenness, diversity, and distances) were calculated including phylogenetic measures like UniFrac [54] at a depth of 3000 sequences per sample. Diversity analysis covered displays and statistics like rarefaction curves, principal coordinate analysis (PCoA), procrustes analysis, biplots, Mantel tests [55], Kruskal-Wallis, bioenv [56], Spearman rank correlations [57], Adonis (conducted in Qiime 1.9.1), ANOSIM, or PERMANOVA tests. Meta-analysis of longitudinal microbial diversity inside built environments was conducted in Qiita [58] and used datasets of the following publicly available Qiita studies: 2192, 10423, 11740, and 12858. All selected studies (including our study as well) targeted the V4 region of the 16S rRNA gene and were processed with the very same Qiita standard workflows (trimmed to 150 bp, used Deblur for denoising of the ASVs and Greengenes 13_8 for closed reference taxonomy assignments). Longitudinal analysis was based on the q2-longitudinal plugin available in Qiime2 [59] and covered calculations of feature-volatility, linear-mixed-effects modeling, pairwise-differences, pairwise-distances, Wilcoxon signed rank tests, and Mann-Whitney *U* tests. Supervised classification and regression of sample metadata was conducted with the q2-sample-classifier plugin [60] with settings for optimized feature-selection and parameter tuning for RandomForest regression and classifications. For this analysis, the dataset was randomly split and 20% of the dataset was removed and kept as the test set. The training set was used to create a learning model predicting class probabilities for each sample by using K-fold cross validation. In the end model, accuracy was calculated by comparing predicted values of the training and test set. Differential abundance and composition of features was determined with balances in gneiss [61], ancom [62], and feature rankings (feature differentials and loadings) available from aldex2 [63], songbird [64], and deicode [65] were partly visualized in qurro [66]. Microbial contributions of different sources and sinks were predicted with SourceTracker2 (https://github.com/biota/sourcetracker2 [67]) at a source and sink depth of 3000 sequences per sample. Potential phenotypes and functions were predicted with PiCrust2 (https://github.com/gavinmdouglas/q2-picrust2 [68]) using the custom tree pipeline and BugBase [69–71]. Further statistics and visualizations were conducted in R [72] using the libraries ggplot2 and streamgraph.

### Data availability

All amplicon raw data is available at the European Nucleotide Archive ENA (EMBL-EBI ERP118380). In addition to raw data, processed data is available in Qiita (study id 12858; https://qiita.ucsd.edu/ [58]).

### qPCR (16S rRNA genes and resistance genes)

The overall microbial load was determined by qPCR of the 16S rRNA gene. Two setups were used to quantify bacteria and archaea separately (primer pair 331f-797r for bacteria [73] and primer pair A806f-A958r for archaea, Supplementary Table S2). For each setup 10 μl of the SYBR Green Supermix (Biorad) contained 5 μl of SsoAdvanced Universal qPCR Kit MM 2×, 0.3 μl each of forward and reverse primers (10 μM), 3.4 μl of PCR-grade water, and 1 μl of template DNA. qPCR runs were then carried out on a Bio-Rad CFX96 thermocycler with the following conditions: initial denaturation at 94 °C for 3 min, followed by 35 cycles of denaturation at 94 °C for 45 s, annealing at 54 °C for bacteria, and 60 °C for archaea for 60 s, elongation at 72 °C for 90 s, and a final elongation step at 72 °C for 10 min. Quantifications of 16 qPCR runs relied on serial dilutions of a cloned 16S rRNA gene of *Escherichia coli* and *Nitrososphaera viennensis*, respectively for bacteria and archaea, into StrataClone vector pscA_AmpKan according to manufacturer instructions. For reliable quantifications, a minimum reaction efficiency of 0.8 and a correlation coefficient above 0.9 as well as clean melting curves were required. Counts in negative and no-template controls were subtracted from actual samples and extrapolated per m^2^. Finally, qPCR counts were displayed as volatility plots including linear regressions with time.

In addition to quantifications of the microbial load, monitoring of antimicrobial resistances was based on the following four selected resistance genes: *blaOXA* (class A beta-lactamase [74];), *int1a* (class 1 integrase [75]), *qacE∆1* (biocide resistance gene, quaternary ammonium compound-resistance [74, 76]), and *tetM* (tetracycline resistance [74, 77]) (Supplementary Table S3). As individual standards were not available, all quantifications were based on relative proportions and serial dilutions (1:10, 1:100, 1:1,000) of the genomic DNA of four cultures (*Acinetobacter* sp., *Escherichia coli*, *Enterococcus*, and *Pseudomonas aeruginosa*) with reported presence of these resistances. qPCR runs were prepared with the STARlet pipetting robot (Hamilton, Germany) for the Bio-Rad CFX384 instrument. PCR conditions were set to initial denaturation at 95 °C for 10 min, followed by 40 cycles of denaturation at 95 °C for 15 s, annealing at 55 °C for 30 s, elongation at 72 °C for 30 s, and a final elongation step at 72 °C for 30 s. All 14 qPCR runs were normalized internally (according to qPCR counts of each individual standard); counts from negative controls and no-template controls were subtracted from actual samples and then extrapolated per m^2^. Similarly, as done for qPCR counts of the 16S rRNA gene, antimicrobial resistances were also displayed as volatility plots with linear regressions.

### Microbial nomenclature

Throughout the manuscript, we refer to the nomenclature assigned by the Silva 128 release. A special case is the genus *Propionibacterium*. We are aware that skin-associated representatives of this genus were renamed in a recent release of the Silva database to the genus *Cutibacterium* [78] that was not available when we performed our analysis.

## Results

### Overview of the microbiome of the built environment and its occupants

Samples were taken from four representative locations (toilet bowl, kitchen floor, desk in one of the bedrooms, desk in the main room) within the confined built environment and from the skin (front torso) of six isolated crew members. Sampling was performed at 27 time points spanning 1 year. Besides amplicon sequencing, all samples plus laboratory controls (*n* = 186 in total) were subjected to quantitative PCR (qPCR) on the 16S rRNA gene to assess the overall bacterial load, and on four representative resistance genes (*blaOXA*, *int1a*, *qacE∆1*, *tetM*) to assess the progression of microbial resistances on skin and surfaces over time.

Along with the samples, 16 types of metadata of environment and crew members were recorded (selected numerical metadata is listed in Supplementary Table S4). The crew was composed of three male and three female members (crewID A-F), with an average age of 30 ± 4 and an average body size of 176 ± 9 cm. During the isolation and confinement, hygiene practices were restricted. On average, crew members showered preferentially on Saturdays, every 5.4 ± 1.8 days (60.67 ± 15.7 times) for about 1 min and 42 ± 47 s. However, individual showering practices differed. For instance, some crew members showered for shorter durations, but more frequently and others showered for longer durations, but only a few times during the isolation period. The diet was mostly composed of dehydrated food, but the crew was allowed to bring beneficial microbes into the habitat, e.g., starters for sourdough bread, tempeh, cream cheese, kombucha, and yoghurt. Toilets were composting toilets. Cyanobacteria (*Anabaena* sp. PCC 7120 and *Chroococcidiopsis* sp. CCMEE 029) were used for research purposes. Part of the kitchen waste was processed in a bokashi composting system.

No direct or real-time contact to other humans except to crew members was allowed, and the extravehicular activities included donning of a mock spacesuit that prevented exposure to open air and direct sunlight (described in [79]). Nine resupply events happened during the isolation period (on days 15, 43, 79, 107, 148, 185, 223, 258, 303, 335). A total of 132 samples were processed before and 43 samples after a resupply event. Temperature was stable over time (mean temperature 18 ± 1 °C). CO_2_ levels were always in the recommended range for indoor environments (400–1000 ppm) with an average of 662 ± 62 ppm.

Denoising of demultiplexed amplicon data with DADA2 resulted in 10,016 unique features (ASVs). In an initial step, we analyzed the processed controls (sampling blanks, process controls, no-template controls), which showed significantly lower microbial Shannon diversity than in actual samples (pairwise Kruskal-Wallis *P* = 1.8 × 10^−4^; Shannon H’ ~ 5.7 vs. 7.0; rarefaction depth of 7850 sequences). Moreover, microbial composition differed significantly between samples and controls, according to weighted UniFrac metrics (PERMANOVA, ANOSIM *R* = 0.42, Adonis *R*^2^ = 0.06, for all three tests *P* = 0.001). To clean the dataset, contaminants were identified from processed controls with decontam [49] and subsequently removed from the dataset. All subsequent analyses were performed with the cleaned dataset, which contained 3,077,780 sequences (median frequency was 17,533 sequences per sample). According to rarefaction curves, sequencing depth was of sufficient quality, as the Shannon diversity metric (H) plateaued at ~ 2500 sequences.

Since we were interested in the characteristics of the microbiome profile of the different sample groups, the dataset was divided into different surface types (crew [skin], built environment) and sample locations (e.g., individual crew members and locations within the facility).

### The overall microbial diversity and composition of biotic (skin) and abiotic surfaces differs significantly

**Fig. 1:**
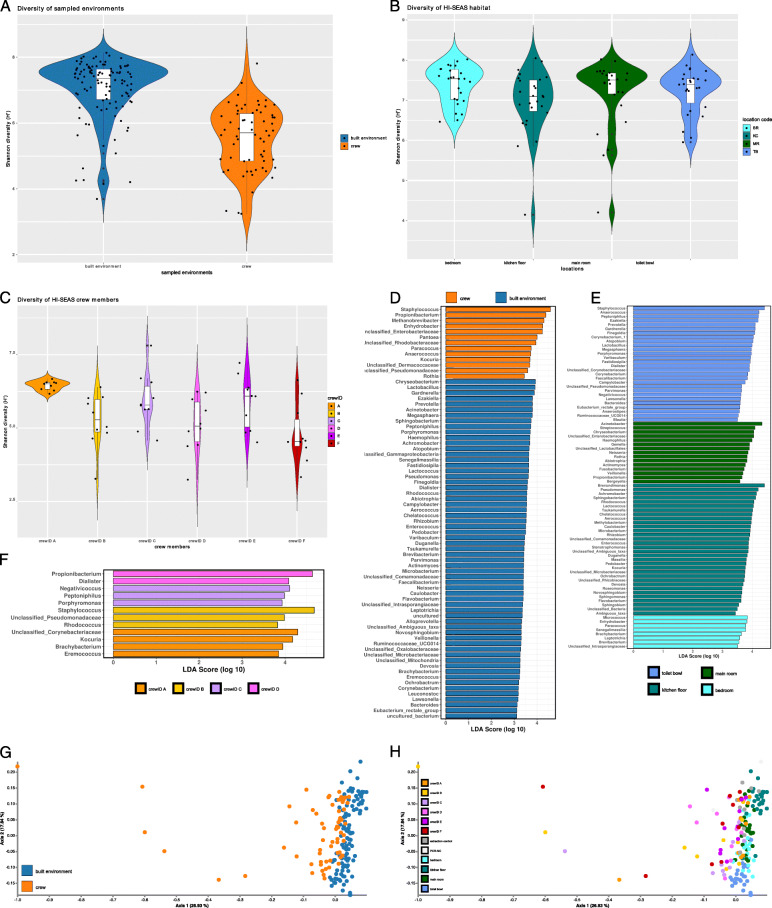
Microbiome profile of crew and built environment samples, all time points included. **a**–**c** Violin plots (kernel probability density of Shannon values, displaying the alpha diversity) including individual data points and a box with median and interquartile range. **a** Sampled surface types: skin of crew and locations of the built environment. **b** Built environment surfaces: BR (desk in bedroom), KC (kitchen floor), MR (desk in main room), TB (toilet bowl). **c** Samples from the individual crew members (**a–f**). **d** Linear discriminant analysis effect size (LEfSe) on samples from crew and built environment. **e** LEfSe on samples from built environment surfaces. **f** LEfSe on samples from individual crew members. **g**–**h** Beta diversity represented by PCoA plots based on weighted UniFrac metrics and a rarefaction depth of 3000 ASVs per sample. **g** Microbial profile of samples from built environment and crew. **h** Microbial profile of the individual crew members and sampling locations of the built environment

In the first step, we compared the alpha diversities (based on Shannon index) of all sample types. Samples from the crew’s skin showed significantly lower diversity than samples from surfaces of the built environment (pairwise Kruskal-Wallis *P* = 7.3 × 10^−16^; mean Shannon H’ ~ 6.2 vs. 7.5) (Fig. 1a). Significant differences were also detected in the diversity index of five crew members (pairwise Kruskal-Wallis of crew member A and B: *q* value 1.6 × 10^−3^; A and D: *q* value 1.3 × 10^−3^; A and F: 7.5 × 10^−3^; C and F: 2.7 × 10^−2^; Fig. 1c).

Notably, the microbial diversity on the crew’s skin varied more (mean Shannon H’ ~ 5.0 in samples from crew member F to ~ 6.7 for crew member A) than that on abiotic surfaces (mean Shannon H’ ~ 7.2 in samples of the kitchen floor to ~ 7.6 in bedroom samples; Fig. 1b, c).

With respect to alpha diversity, the built environment surfaces showed only significant differences between bedroom and kitchen floor samples (pairwise Kruskal-Wallis *q* value 4.5 × 10^−2^; Fig. 1b), while richness was variable (Supplementary Fig. S3). Alpha diversity was also significantly different according to type of surface material (plywood vs. polymer; pairwise Kruskal-Wallis *q* value 4.5 × 10^−2^; Supplementary Fig. S4).

Beyond alpha diversity, the microbiome profile of samples from built environment surfaces was significantly different from that of crew skin samples (weighted UniFrac distances, PERMANOVA *q* value = 3 × 10^−3^; ANOSIM *R* = 0.3, *P* = 3.3 × 10^−3^; Adonis *R*^2^ = 0.15, *P* = 1 × 10^−3^) and samples clustered separately in PCoA plot analysis (Fig. 1g, h). Further significant differences were found between all four locations of the HI-SEAS habitat (ranging from *q* values of 1.8 × 10^−3^ to 3.3 × 10^−3^, PERMANOVA pairwise testing; ANOSIM *R* = 0.14 to 0.85, highest for kitchen floor vs. toilet bowl; Adonis *R*^2^ = 0.33). However, the microbial composition was not significantly different between individual crew members despite highly explained variability along PCoA axis 1, indicating dynamic changes of microbial composition on skin samples over time (see below).

Supported by extensive metadata analysis, the factors time (*P* = 0.04, Spearman rank correlation of Shannon diversity with time) and sampling location (*P* = 9.4 × 10^−19^, Kruskal-Wallis test of all groups, see above for more details) were identified to have a significant impact on microbial diversity and the microbial profile, whereas the microclimate of the habitat revealed no significant influence.

Metadata predictions based on Random Forest classifiers and regressors showed high overall accuracy estimates of 95% for the sampling environment (skin samples vs. built environment samples), and the day of sampling (*R* = 0.77, *P* = 2.3 × 10^−8^). Thus, our subsequent analyses focused on the impacts of time and sampling location.

### Each surface was characterized by a specific set of microbial signatures, which can be predicted with high accuracy

As selected surfaces of the built environment (desk in a bedroom, kitchen floor, desk in the main room, toilet bowl) and the crew’s skin showed a significantly different composition (see below), we were interested in a detailed analysis of the characteristic features.

Overall, the skin samples were characterized by high abundance of *Staphylococcus*, *Propionibacterium*, *Enterobacteriaceae*, *Enhydrobacter*, and *Methanobrevibacter* signatures (LEfSe analysis, Fig. 1d), whereas the built surfaces were characterized by the presence of *Chryseobacterium*, *Lactobacillus*, *Gardnerella*, *Prevotella*, and *Acinetobacter*.

Indicative microbial signatures were identified for the toilet bowl (*Staphylococcus*, *Anaerococcus*), the main room desk (*Acinetobacter*, *Streptococcus*), the kitchen floor (*Brevundimonas*, *Achromobacter*), and the bedroom desk surface (*Enhydrobacter*, *Micrococcus*; Fig. 1e).

As observed for habitat surfaces, individual crew members revealed typical microbiome profiles with *Propionibacterium* being indicative for crew member D, *Peptoniphilus* for crew member C, *Staphylococcus* for crew member B, and *Kocuria* for crew member A (Fig. 1f). Remarkably, accuracy of metadata prediction based on RandomForest classifications was possible for certain individuals (e.g., crew member D with 100%) or distinct surfaces of the built environment (e.g., kitchen floor and the toilet bowl both 100% accuracy) (Supplementary Figure S5).

### Microbial diversity on skin increased during the isolation over time

**Fig. 2:**
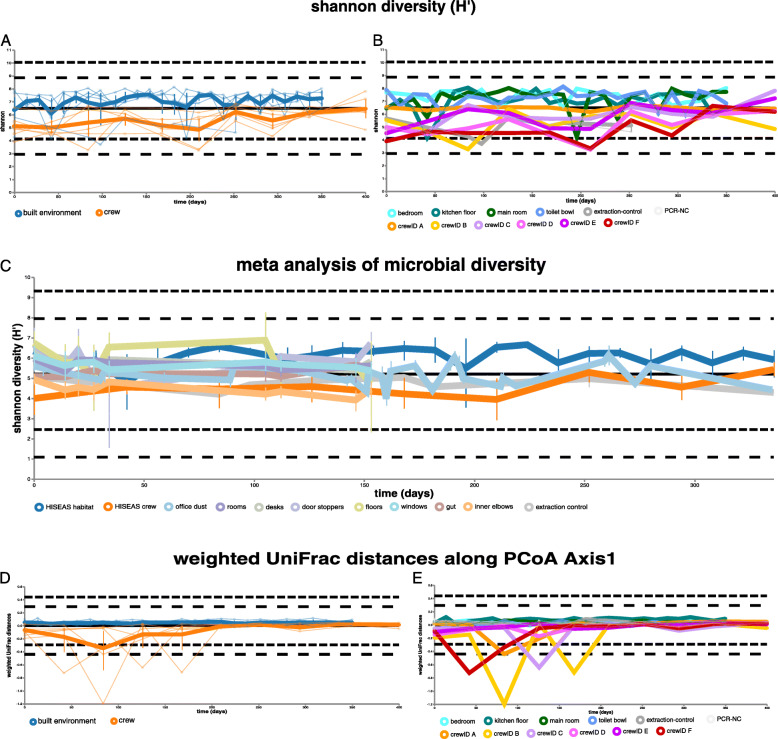
Longitudinal development of diversity and composition. **a** Volatility analysis of Shannon diversity resolved to sample type (built environment and human skin). **b** Volatility analysis of Shannon diversity resolved to individual crew members and sampled locations within the HI-SEAS habitat. **c** Meta volatility analysis of longitudinal microbial diversity of samples taken within the HI-SEAS habitat and inside other built environments. **d** Volatility analysis along PCoA axis 1 showing weighted UniFrac distances for different sample types (built environment and human skin). **e** Volatility analysis along PCoA axis 1 showing weighted UniFrac distances for individual crew members and sampled locations within the HI-SEAS habitat

Overall, the longitudinal microbial diversity in samples from skin showed a steady increase over time (mean Shannon *H*′ 4.9 to 6.4), whereas the increase in microbial diversity on built environment surfaces was lower (mean Shannon *H*′ 6.4 to 7.3). The microbial diversity from built environment surfaces was subject to greater fluctuations throughout the time period (Fig. 2a). This observation, however, could be due to a higher number of analyzed built environment samples. Increasing microbial diversity on skin was also confirmed by linear mixed effect models which tested whether Shannon diversity changed over time in response to the sampling locations (Supplementary Fig. S6). An increase in microbial diversity on skin was observed for most crew members (C, D, E, and F; mean Shannon *H*′ 5.1 to 6.5; highest increase for individual C from 5 to 7.8). However, almost no change was visible for individual A, and a slight decrease was observed for individual B (mean Shannon *H*′ 5.5. to 4.8; Fig. 2b).

The microbial diversity on different locations inside the HI-SEAS habitat changed as well (Fig. 2b). The largest fluctuations were detected for samples of the main room, and a slight increase in microbial diversity was visible for all locations apart from the toilet bowl. In the latter case, microbial diversity decreased by 1 log (mean Shannon *H*′ 7.9 to 6.8), possibly due to more rigorous cleaning procedures.

Other metrics describing the alpha diversity of all samples, such as richness (92 to 213.67) and phylogenetic diversity estimates (7.6 to 14.7), followed a similar pattern, while Pielou’s evenness remained constant over the entire isolation period (0.8 to 0.84; Supplementary Fig. S7 and Supplementary Fig. S8).

Temporary dynamics of microbial diversity were investigated by pairwise difference comparisons of samples from individual time points. Significant differences were only evident between day 210 and day 252 for skin samples (Kruskal-Wallis test for multiple groups, *P* = 0.02) and between skin and built environment surface samples (Mann-Whitney *U* test, *q* value = 0.03).

Furthermore, correlating patterns of microbial diversity were analyzed by Spearman rank correlations. After false discovery rate (FDR) correction, significant positive correlations of microbial diversity were only evident between crew members C and E (*q* value = 0.05^−4^, rho = 0.9).

Observations of microbial composition followed a similar pattern as described for microbial diversity. Hence, composition of skin samples (weighted UniFrac distances) changed to larger magnitudes than those from built environment surfaces along PCoA axis1 (Fig. 2d). Largest shifts on crew’s skin were visible between day 0 and day 210 (with a maximum at day 84 of − 0.3) (Fig. 2d), especially for crew member B. In contrast, almost no changes along PCoA axis 1 were visible for crew member D and E (Fig. 2e).

Pairwise distance comparisons of microbial composition (weighted UniFrac distances) at individual time points showed significant differences between day 84 and day 126 (Kruskal-Wallis test for multiple groups, *P* = 0.03), and between skin and built environment surface samples (Mann-Whitney *U* test, *q* value = 0.04).

### Comparison with other built environment studies indicates an atypical increase in skin diversity under isolation

For a suitable evaluation of our observations, we performed a meta-analysis of longitudinal microbial diversity patterns inside different built environments. This analysis (see “Material and methods” section for more details) covered more than 3400 samples and ten different sample types (front torso skin, confined habitat surfaces, office dust, room surfaces, desk surfaces, door stoppers, floors, windows, fecal samples, and skin samples from the inner elbow) from four longitudinal studies inside the built environment and were all processed in the same way to allow for proper comparisons. Public studies from Qiita were selected based on three criteria: first they had to be longitudinal, second they had to be conducted in a built environment setting, and third they had to cover samples from human sources beside built environment surfaces. According to these criteria, we included a longitudinal analysis of microbial interactions between humans and the indoor environment [80], a longitudinal assessment of the influence of lifestyle homogenization on the microbiome of United States Air Force Cadets [81], and a study which identified geography and location as the primary drivers of office microbiome composition [82]. Our meta-analysis confirmed that microbial diversity progressions in the HI-SEAS habitat were exceptional. While all other sample types showed a decrease in microbial diversity, only samples from the human gut of US air force cadets (mean Shannon *H*′ 5.1 to 5.5) and skin samples of the HI-SEAS crew (mean Shannon *H*′ 4.9 to 5.4) showed a steady increase over time (Fig. 2c).

### Microbial dynamics during isolation was driven by specific taxa

For a higher resolution of microbial composition in skin and built environment samples over time, the isolation period was grouped into four phases (phase 1: days 0–84, phase 2: 84–210, phase 3: 210–294, and phase 4: 294–336). To get insights into microbiome evolution after isolation, skin samples from the post-mission control (day 400) were also studied.

**Fig. 3:**
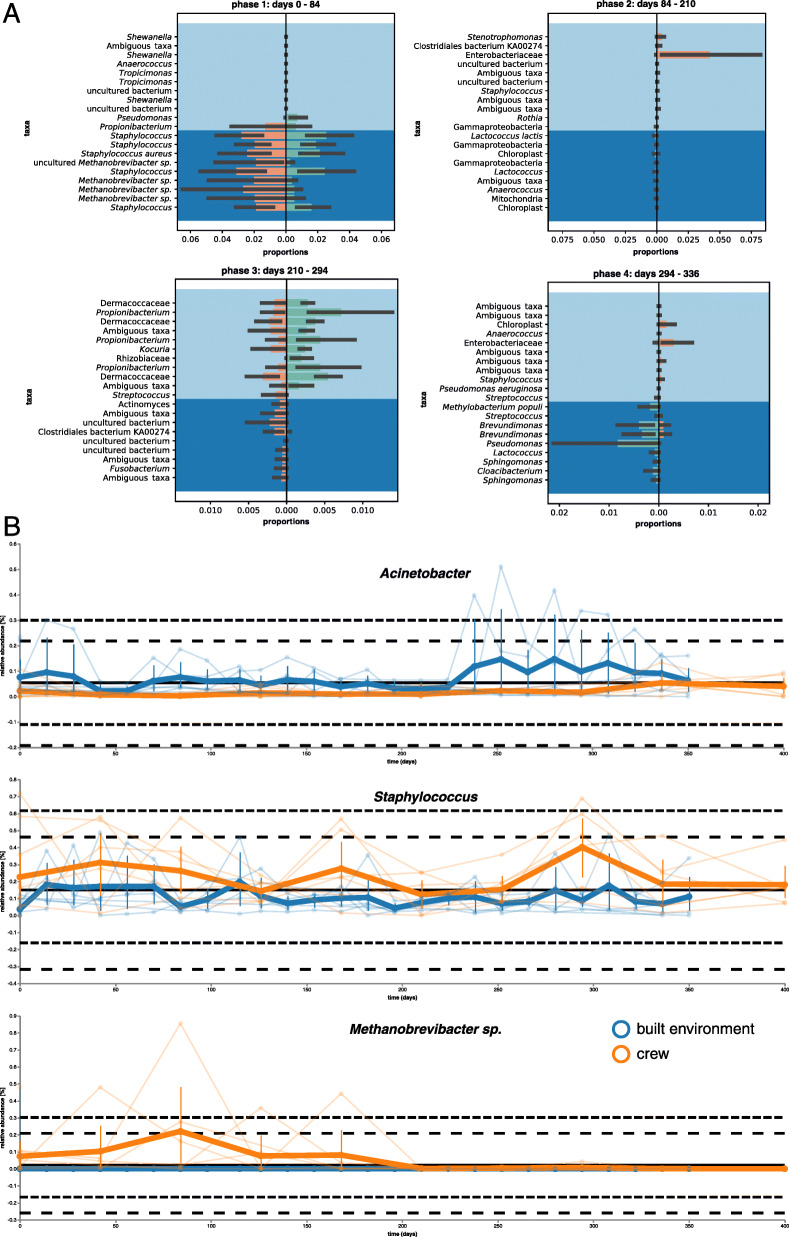
Microbial dynamics in distinct phases and for representative features. **a** Proportion plots of differential feature abundances using balances in gneiss on genus level grouped into four distinct phases. The proportion plot shows taxa of the crew and the built environment, which could be responsible to explain the differences between the earlier and the later sampling event in each phase (green and orange bars). Differential numerator taxa are grouped to the top (background color in light blue) and differential denominator taxa are grouped to the bottom (background color in dark blue). **b** Volatility analysis based on linear regression models with time of *Acinetobacter*, *Staphylococcus*, and *Methanobrevibacter* sp.

According to differential abundance analysis of all samples using balances in gneiss (Fig. 3a), higher proportions were visible for *Staphylococcus*, *Propionibacterium*, and *Methanobrevibacter* (phase 1). Between day 84 and day 210 (phase 2), only *Stenotrophomonas* and unclassified Enterobacteriaceae showed higher proportions at the latter time point. Later on, unclassified Dermacoccaceae, *Propionibacterium*, *Kocuria*, and unclassified Rhizobiaceae showed increasing proportions, while *Streptococcus* and *Fusobacterium* showed decreasing proportions (phase 3). During phase 4, *Methylobacterium populi*, *Streptococcus*, *Brevundimonas*, *Pseudomonas*, *Lactococcus*, *Sphingomonas*, and *Cloacibacterium* revealed lower proportions than unclassified Enterobacteriaceae and *Staphylococcus*.

After the isolation period, increasing proportions of *Acinetobacter*, *Propionibacterium*, *Rhizobium*, and *Methylobacterium populi* were prevalent on the skin of the crew, while signatures of *Pseudomonas*, *Corynebacterium*, or unclassified *Intrasporangiaceae* decreased (Supplementary Fig. S9).

### Dynamics of representative skin, GIT/UGT, and environment-associated microbial taxa

In a next step, we selected 15 microbial genera and families which were indicative of either skin (*Acinetobacter*, *Staphylococcus* [*aureus*], *Brevundimonas*, *Kocuria*, *Propionibacterium*, *Streptococcus*, *Kytococcus*, Dermacoccacae), gastrointestinal/urogenital tract (*Gardnerella*, *Lactococcus*, *Methanobrevibacter*, *Faecalibacterium*, Enterobacteriaceae), or environment and water (*Pseudomonas*, *Enhydrobacter)*, to assess the dynamics of those microbial signatures. Our feature selections were supported by higher rankings in differential abundance tests based on gneiss, aldex2, and songbird, and feature loadings based on deicode. Grouping these representative features into the categories skin, GIT/UGT (gastrointestinal/urogenital tract), and environmental was based on empirical data from literature [10].

Representing the skin microbial taxa, *Acinetobacter*, despite being recognized as a typical skin microbial taxon*,* showed higher relative abundances on built environment surfaces (especially in the main room and bedroom). Crew member E showed over proportional prevalence at the beginning and together with crew member A also at the end of the isolation period (Fig. 3b and Supplementary Fig. S10).

*Staphylococcus* [*aureus*] was mainly present on human skin (crew members D, E, and F), but has also been detected on surfaces inside the habitat. (Fig. 3b and Supplementary Fig. S11). *Brevundimonas* was clearly associated with the kitchen surfaces (Supplementary Fig. S12) and showed higher proportions on the toilet bowl (between day 70 and day 84), the main room (between day 238 and day 252), and on the skin of crew member F between day 238 and day 252 and again between day 294 and day 336. Relative proportions of *Kocuria* were clearly correlated with time by linear regression models and showed the highest value for importance (0.3) (Supplementary Fig. S13). While crew members A and E showed a higher prevalence of *Kocuria* right from the beginning, built environment surfaces as well as crew members B and C showed higher proportions only later on. In contrast, *Propionibacterium* did not manifest itself on built environment surfaces and could only be recovered from other skin samples over time (Supplementary Fig. S14). On the other hand, signatures of *Streptococcus* could not be linked to a defined human source and established itself on bed and main room surfaces (Supplementary Fig. S15). *Kytococcus* was associated with crew members A and D at the beginning (Supplementary Fig. S16). Later on, only single events of high proportions were visible on the skin of crew member B or sampled bedroom surfaces. Dermacoccaceae were regularly retrieved from skin and built environment surfaces with highest proportions in samples from the bedroom and from crew member D at the end of the confinement period (Supplementary Fig. S17). After *Kocuria*, Dermacoccaceae showed the highest importance (0.1) in linear regression models.

As a representative of the GIT/UGT, *Gardnerella* showed a consistent presence despite varying proportions on the surface of the toilet bowl (Supplementary Fig. S18). Likewise, signatures of *Lactococcus* revealed a single peak on the kitchen floor after day 50, but could not be detected on the skin of any crew member (Supplementary Fig. S19). In contrast to *Faecalibacterium* (Supplementary Fig. S20), *Methanobrevibacter* were not consistently recovered from the toilet bowl, but were clearly associated with some of the crew members (Supplementary Fig. S21). As biplot analyses identified Euryarchaeota (in particular *Methanobrevibacter* sp.) as the main reason for compositional changes around day 84, this genus was analyzed further. In general, signatures of *Methanobrevibacter* were highly associated with the human crew within the first 210 days (Fig. 3b and Supplementary Fig. S22). However, these signatures were not common on built environment surfaces and were only observed on the toilet bowl and the kitchen floor on day 115 and on day 224. This pattern was different from that of other archaeal lineages (Euryarchaeota, Thaumarchaeota, and Woesarchaeota), which showed scattered peaks on built environment surfaces but not in skin samples. Enterobacteriaceae showed only single events of prevalence on the toilet bowl and were mainly associated with skin samples of crew members D, E, and F between day 100 and day 250 (Supplementary Fig. S23).

Despite representing an environment-associated taxon, *Enhydrobacter* was present on all crew members to varying proportions and was regularly detected in samples from the toilet bowl (Supplementary Fig. S24). Signatures of *Pseudomonas* were observed in skin samples from crew members B and D, followed by a detectable increase on the kitchen floor and further on the skin of crew members E and F (Supplementary Fig. S25).

### Shared occupancy influences the microbiome composition and function of the crew skin and abiotic surfaces

**Fig. 4:**
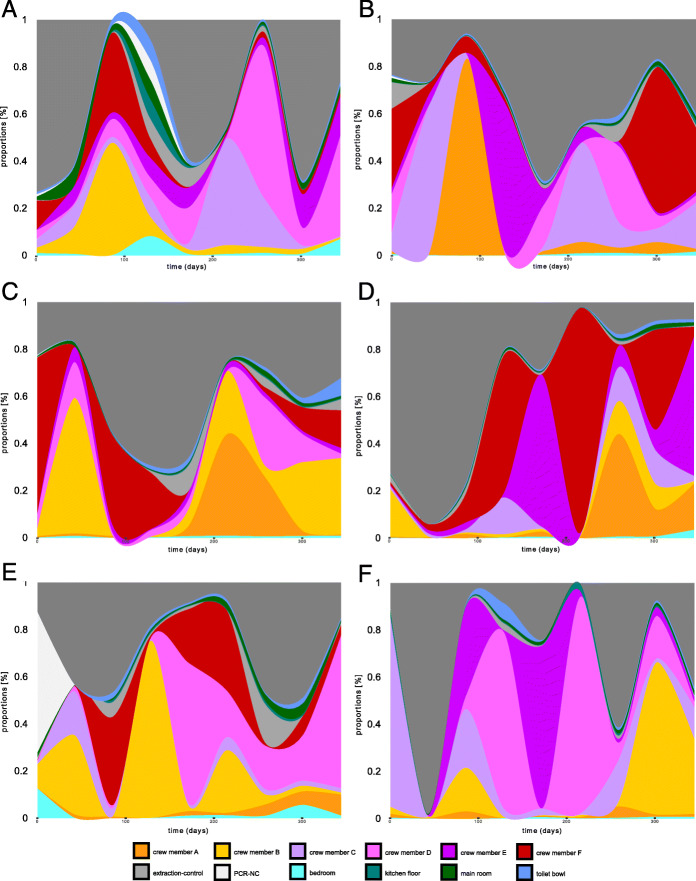
Source tracking of microbial signatures according to SourceTracker2. Panels **a–f** show respective crew members A to F as a sink of microbial dispersal

Source tracking of microbial signatures with Sourcetracker2 identified human skin as the main source of microbial dispersal. Noteworthy, the intensity of microbiome exchange was heterogeneous among the possible pairs of crew members. In more detail, crew member F showed the highest interactive profile (13.4%) of all crew members. This was also supported by redundancy analysis (RDA) revealing pairwise microbial exchange for two pairs of crew members as significant parameters on respective skin microbiome profiles (*P* = 0.002 RDA). Sampled locations of the built environment played only a minor role in overall microbial dispersals. Maximal microbial contributions on the crew reached only proportions of 1.2% in case of the main room. Interestingly, crew gender showed different microbial associations for bedroom and the kitchen floor samples. Nevertheless, microbial interaction profiles were highly person-specific as well as dynamic over time. Overall, trends were difficult to delineate (Fig. 4).

**Fig. 5:**
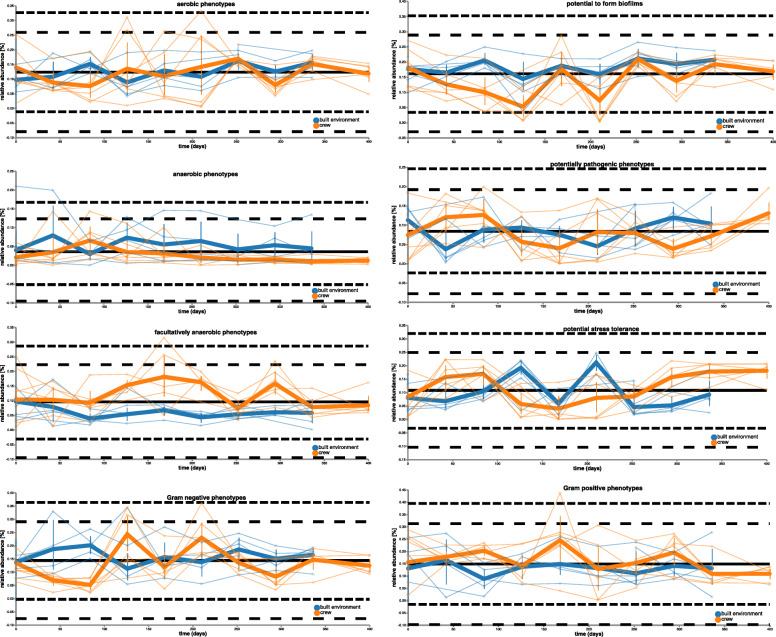
Linear regression with time of predicted phenotypes from BugBase shown as volatility plots (sorted according to regression with time)

Further on, we were interested in whether microbial profiles and interactions between crew members and surfaces inside the HI-SEAS habitat had an impact on potential phenotypes predicted with Picrust 2 and BugBase. According to these predictions, most phenotypes showed higher proportions (e.g., potential mobile elements, potential pathogens, potential stress tolerance, and especially facultative anaerobes) or recurring peaks (Gram-positive and Gram-negative phenotypes and aerobes) on samples from the crew’s skin. However, the potential to form biofilms showed a constant maximum in samples retrieved from the kitchen floor (global median 0.18, maximum 0.27, cumulative average decrease/increase − 0.25/0.24) and anaerobes were increasing on the toilet bowl (minimum 0.06, maximum 0.21) while decreasing on human skin (0.02 to 0.01). Interestingly, potential pathogens showed an anti-cyclic pattern of samples from the built environment versus samples from the crew (Fig. 5).

### Fluctuation of microbial quantity correlates with the presence of certain antimicrobial resistance genes and microbial phenotypes

**Fig. 6:**
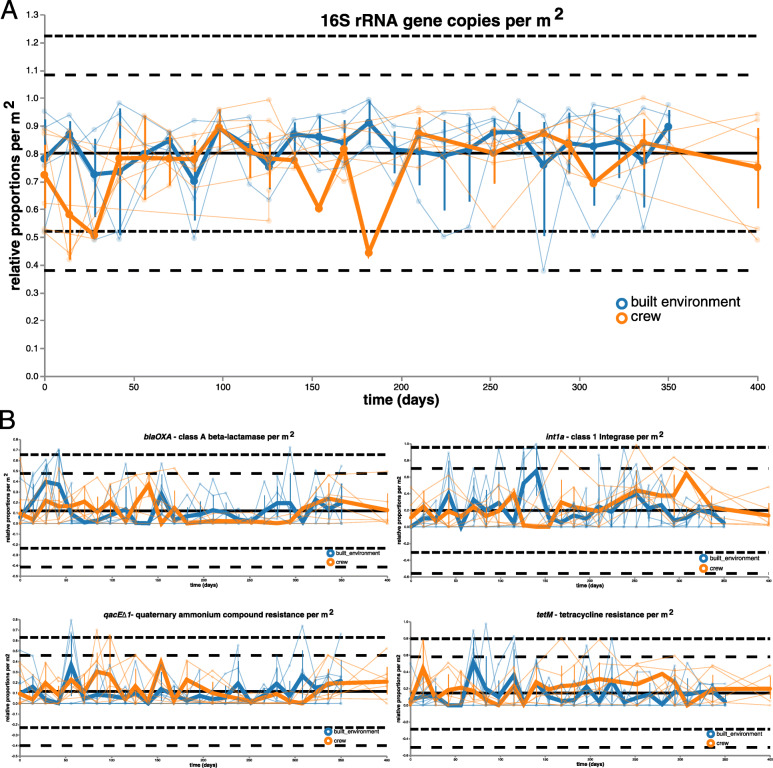
Overall bacterial load and abundance of selected antimicrobial resistances. **a** Volatility analysis of bacterial 16S rRNA gene copies per m^2^ according to qPCR. **b** Linear regression volatility analysis of selected resistance gene copies per m^2^ according to qPCR

Quantitative PCR was used to assess bacterial and archaeal abundance, and its dynamics inside the HI-SEAS habitat and the skin of its isolated crew members. As observed for microbial profiles, bacterial abundance changed to a larger extent for samples from the crew’s skin than from built environment surfaces. In general, two main phases of differing bacterial load could be determined. In the beginning (days 0–42) and between days 126 and 210, bacterial abundance on human skin was much lower than on selected locations of the built environment (respective mean difference for the two phases: 12.5 and 22.5%). The largest dynamics were observed around day 28 and day 182 (change in relative proportions by 43%; Fig. 6a). On the other hand, archaeal abundance peaked around day 84 (82%) but varied to much lesser extents, especially in the mid-term of the isolation period (Supplementary Fig. S26).

In addition, four markers for antimicrobial resistance (*blaOXA*—class A beta-lactamase, *int1a*—class 1 integrase, *qacE∆1*—biocide resistance gene, quaternary ammonium compound, and *tetM*—tetracycline resistance) were selected to analyze dynamics of microbial resistances in a quantitative way. *TetM* was most predictive for the factor time (importance = 0.2) and was constantly more abundant in skin samples between day 140 and day 294. Interestingly, beta-lactamases showed the opposite pattern, with lowest proportions between day 140 to day 294. *Int1a* gene abundance was highly dynamic over the whole time frame (highest global variance of 0.06) and showed peaks on built environment locations (toilet bowl and kitchen floor) on day 140, but also on human skin (especially crew members C and D) on day 308. Highest and lowest abundances of *qacE∆1* regularly alternated between samples of the built environment and from human skin. Nevertheless, all four targeted resistance genes showed high dynamics and potential transfer between skin and the built environment (Fig. 6b).

Eighty-nine taxa on species level could be positively correlated by Spearman rank correlations with 16S rRNA gene abundance (for instance *Chryseobacterium q* value = 1.5 × 10^−8^, *R* = 0.57; *Pseudomonas fragi q* value = 1.6 × 10^−8^, *R* = 0.56; *Megasphaera q* value = 7.5 × 10^−8^, *R* = 0.54), while only a few taxa showed significant negative correlations (*Ralstonia q* value = 1.8 × 10^−4^, *R* = − 0.39; *Tepidimonas q* value = 0.01, *R* = − 0.26). On the contrary, potential significant correlations of taxa with selected antimicrobial resistance genes could not be verified by multi-hypothesis testing using FDR correction of significant *p* values.

Finally, predicted phenotypes were correlated both with each other and with obtained quantitative information (16S rRNA gene copies and selected resistance genes). While all quantitative data could be significantly positively correlated with each other (especially class A beta-lactamases with biocide resistance of quaternary ammonium compounds; *q* value = 1.5 × 10^−13^, rho = 0.66, and biocide resistance of quaternary ammonium compounds with tetracycline resistance;*q* value = 2.9 × 10^−7^, rho = 0.5), comparisons of the qualitative information showed both positive and negative correlations. Significant positive correlations were evident between aerobes and potential biofilm formers (*q* value = 8.9 × 10^−11^, rho = 0.60), as well as potential pathogens and stress tolerance (*q* value = 4.7 × 10^−12^, rho = 0.63). On the contrary, significant negative correlations were observed between aerobes and facultative anaerobes (*q* value = 1.0 × 10^−14^, rho = − 0.67), as well as between potential biofilm formers and Gram-positives (*q* value = 1.1 × 10^−7^, rho = − 0.51). However, significant correlations between quantitative and qualitative measures were scarce. Only the overall bacterial load (16S rRNA gene copy numbers) showed significant positive correlations with anaerobes (*q* value = 0.002, rho = 0.32) and significant negative correlations with aerobes (*q* value = 0.01, rho = − 0.26).

## Discussion

Human well-being is inseparably linked with its microbiome. Thus, the dynamics of the microbiome in and around a human being is subject of research for the preparation of human long-term spaceflight and settlement in remote locations such as a future Martian outpost [7]. For such studies, Earth-based models are indispensable including a monitoring of the longitudinal microbial dynamics of an isolated crew with its confined environment beside social analysis of team cohesion and performance. Numerous suitable model environments were investigated in the past (besides the one studied herein), but greatly differed in terms of setup and study design. For instance, the Concordia research station in Antarctica comprised separate buildings, accommodated 16–32 occupants during the sampling period, and was microbially monitored for 365 days [43]. The US inflated lunar/Mars analog habitat (ILMAH) provided 300 m^3^ space for three occupants and was investigated for 30 days [44]. Another example is the Mars500 habitat located in Moscow, Russia, which included four modules with a total volume of 550 m^3^ and housed six participants for 520 days [41].

All isolated and confined built environment (ICE) models represented a unique testbed for assessing the microbial interaction of an isolated crew inside a confined built environment. In contrast to other studies in the microbiome of built environments (MoBE) field, its reduced set of potential environmental variables that could drive the microbiome in a longitudinal context allows distinct assumptions about where microbial signatures originated, as well as when, where and why they were transferred through time and space [83].

Unexpectedly, our study revealed a highly dynamic skin microbiome of the HI-SEAS crew despite its isolated and confined setup. In contrast to the study by Sharma and co-workers [81] in which longitudinal homogenization of microbial composition was visible *ab initio*, we observed a retarded longitudinal homogenization between skin and built environment samples only after 210 days. Nevertheless, traces of the skin microbiome were clearly visible on the studied surfaces and as described before occupancy was identified as the major microbial source [9, 84–86]. Compared to microbial interaction profiles from human skin, building surfaces seem to play a more passive and subordinated role for spreading and dispersal in this habitat and confirmed again that humans dominate microbial communities on indoor surfaces [87, 88]. Accordingly, the kitchen floor surface stood out with its low microbial diversity. This observation could be associated with the low interaction frequency of this specific location (floor surface) in contrast to the regularly touched desk or toilet surfaces in the habitat.

In contrast to other isolation experiments like the Mars500 study [41] in which a significant decrease in microbial diversity was observed, the microbial diversity on the HI-SEAS surfaces remained rather constant and even increased in samples from crew skin. Confirmed by our meta-analysis based on four selected longitudinal studies from built environment settings, increasing microbial diversity on human skin and built environment samples was not reported in offices or homes [80, 82]. However, this observation could be the result of a human occupancy in confined and in more isolated built environments as this pattern was also observed in samples from crew members at the ISS (International Space Station) for samples of the forearm and gut [89] or in fecal samples during the cohabitation of US Air Force cadets [81]. Crew members C, D, E, and F started with a relatively low range of microbial diversity on their skin compared to representative studies of the skin microbiome [90, 91]. This rather low microbial diversity might result from extensive personal hygiene cleaning procedures before entering the HI-SEAS habitat with a limited allowance of daily hygiene procedures.

The sources of microorganisms are not completely clear; the steady increase on the crew’s skin could result from interactions with the higher microbial diversity present in their habitat (refer to Fig. 2a, b), regular resupply events, sanitary complications, and elaborate cleaning of the composting toilet (for details see below) or dispersal of microbial food supplements used to ferment food. In addition to these potential microbial sources, the altered hygiene regime during isolation has probably contributed to an increase or stabilization of skin microbiome composition. The most stable skin microbiome was observed for a crew member, who applied extremely short showers (average duration of 40 s; Supplementary Table S4), compared to the other crew members (overall average showering time of 102 s), which revealed a more fluctuating microbial profile. Another impact of hygiene regimens on the skin microbiome was indicated by the higher abundance of quaternary ammonium compound resistance genes on the skin of individual crew members. This observation reflected the application of disinfecting cleaning wipes (which contained such compounds) on the crew’s skin in-between the showering events.

The most striking observation was the delayed longitudinal homogenization and very dynamic developments of the skin microbiome in the first half of the isolation period (days 0–210). These unstable profiles correlated with complications of the composting toilet, which required a manual cleaning and emptying rotationally by all the crew members. Dispersal of gut/urogenital-associated microorganisms from the composting toilet might have even been supported by the use of a fan to reduce the odor. An obvious indicator species of these complications was *Methanobrevibacter*. As verified by biplots, specific filtering of the dataset and significant correlations of quantitative data (bacterial and archaeal abundance) with an anaerobic predicted phenotype, this archaeon of the human gut explained most of the dynamics in this timeframe and delayed the expected longitudinal homogenization process of its microbiome. *Methanobrevibacter* represents the most common archaeal genus of the human gut and has an important role at the end of the intestinal food chain where it facilitates the fermentation process of bacteria by removing hydrogen from the system (an overview is given in [92]. Obviously, the increased prediction of anaerobic phenotypes correlated very well with the increased signatures of *Methanobrevibacter* in the same timeframe. Later on, the signatures of *Methanobrevibacter* increased relatively on samples of the toilet bowl while decreasing on human skin as soon as the toilet issue was fixed. Most of the other predicted phenotypes showed higher relative proportions and dynamics on skin samples, which might be due to the reported temporal and personalized variability on human skin samples [90, 93]. In addition, the anti-cyclic pattern of potential pathogens might picture regular transfer events between skin and desk surfaces in the habitat, which were described before [81].

Besides the remarkable traces of gut-associated archaea, other selected bacterial indicator species were helpful to interpret microbial dynamics in the HI-SEAS habitat. Hence, most skin-associated bacteria like *Staphylococcus aureus*, *Brevundimonas*, *Kocuria*, *Propionibacterium*, *Streptococcus*, *Kytococcus*, and Dermacoccacae could be easily traced in the habitat, were most frequently exchanged with the desk surface in the bedroom, and were transferred more likely between crew members who also had close physical interaction with each other. Their presence on different built environment locations was often transient and higher proportions were usually detected in samples from the crew. This observation was also confirmed by source tracking analysis, which showed higher microbial transfer between crew members A and D, B and C, or E and F, or low microbial transfer between crew members A and F, or C and E. This microbial transfer pattern corresponds to the reported interaction preference of male with female crew members during the isolation period. These results underline the important impact of the personal microbial cloud [94], the importance of direct physical interaction [80, 95, 96], as well as cohabitation of crew members [97] and the low microbial input from selected built environment locations [88] to the overall microbiome of the HI-SEAS habitat.

*Propionibacterium* could act as an example for the low input of the built environment microbiome, since this bacterium was only recovered from skin samples and was never observed on surfaces of the HI-SEAS habitat. This might be due to its strict anaerobic lifestyle, that seems to prefer the oxygen depleted pores of human skin and much less those relatively smooth oxygen rich building surfaces and materials.

However, an opposite profile was observed for signatures of *Acinetobacter*. Although described as a skin-associated microbe [98], *Acinetobacter* established itself much better in various locations of the main room and bedroom. As this bacterium is also frequently detected on surfaces of public and private buildings, as well as hospitals or cleanrooms [99–102], it is tempting to speculate whether *Acinetobacter* could be perceived as an indicator species for confined built environments and to a lesser extent only as a skin-associated bacterium.

Peaks of signatures from *Lactococcus* on the kitchen floor might be due to its usage in fermenting yoghurt, cream cheese (*Lactococcus lactis* subsp. lactis and cremoris), tempeh, or kombucha. The idea of using microbial cultures as part of the food supply, or entire artificial ecosystems for life support [28, 103], is of particular importance in isolated and confined built environments (ICE) and might be key to colonizing another celestial body in the solar system. Nevertheless, the exact composition of commercially available products must be well-characterized to mitigate potential risks for the crew.

Although a number of metadata about health and medication of the crew were obtained, the information was not precise enough for correlations with the microbiome. Crew members provided medication data, but only two reported treatments could be associated with increases in tetracycline resistance and bacterial abundances, potential pathogens or stress tolerance. Hence, in contrast to the study by Abeles and co-workers [104] who could follow specific treatment courses in the microbiome, our dataset was not comprehensive enough to make such assumptions. On the contrary, recordings of the microclimate produced numerous values, but was rather constant and did not represent a major driving factor of the microbiome (only up to 9%) as investigated by bioenv analysis.

The presented study has limitations and serves only partially as a basis for meaningful recommendations for future attempts to sustain a safe microbial environment in a human outpost on the Moon or Mars. First of all, no baseline (i.e., before the start of the isolation experiment) of the indoor and skin microbiome was defined. In addition, information on all microbial sources were limited and only one body site was selected for monitoring potential microbial transfer with the environment. Additional human samples would have been supportive to draw conclusions about an individual’s health status (e.g., fecal samples) or draw detailed conclusions of frequently touched surfaces inside the habitat (e.g., hand samples). In general, the entire infrastructure and setting mimicked only partially a real extraterrestrial, crewed mission. This impacts for instance the availability of water for personal hygiene, or selected sanitary products.

Nevertheless, the study benefits from its defined confined setup with limited amount of confounding environmental variables, a defined set of occupants, the deduced prediction and tracking potential of microbial transfer in a remarkable level of detail, the correlation of qualitative and quantitative microbial and resistance data, and our pre-informed knowledge of microbial hotspots on desks, in bedrooms, or the toilet bowl on ICE locations from the Mars500 project [41].

## Conclusions

From a microbiological point of view, our methods were not suitable to determine an elevated risk of infection or transfer of antimicrobial resistant pathogens to crew members. However, due to recent information from the ISS [24], we consider this risk as extraordinarily low. Nevertheless, although the risk was deemed to be low, the need for monitoring microbial dynamics inside isolated and confined habitats became obvious, in order to understand the impact of special events like the contamination caused by the composting toilet. Moreover, baselines of the microbiome inside the habitat, its crew, and post sampling events would greatly improve evaluations of the impact of confinement on the crew and habitat surfaces. Most comparable studies, either ground-based like Mars500 [41, 105] or in space aboard the ISS [24, 89, 106], showed a similar picture of longitudinal homogenization, composition, and diversity of microbiomes.

However, in many cases, these studies suffered from a lack of appropriate controls, or baselines, which are a central point of management of metadata or interpretations. For future experiments, it is key to determine a standard operating procedure regarding sampling intervals, methods suitable for microbial monitoring, distinct knowledge on pre- or probiotics used for food production, stabilization of the gut microbiome or even personalized collections for autologous fecal microbial transplants in case of worrisome developments leading to a microbial dysbiosis. Hence, future ICE missions in preparation for crewed mission to the Moon or Mars in the upcoming decades should emphasize on actual tests on a microbial warning system that could be based on automatic sampling technologies and predictive models comparing expected and true microbial compositions in the habitat and its crew. Furthermore, manipulations of the microbiome would be essential to stress the microbiome by different levels of desired (reduce potential pathogens and technophilic microorganisms) and unintentional perturbations through defined cleaners and antibiotics, and following restorations of a beneficial community on the surfaces of the habitat, as well as different body sites including the crew’s gut microbiome. One key question for future-related studies is how diverse a microbiome needs to be and which actual composition it needs to have on surfaces, and on and in the human body, to be self-sustaining over longer timeframes, or how it can be stabilized and restored after critical perturbations. The answers to these questions are a crucial prerequisite for the planning of future human long-term missions in space or on other celestial bodies in our solar system.

## Supplementary Information

**Additional file 1: Supplementary Table S1.** 16S rRNA gene primers used for generating the amplicons for Illumina MiSeq sequencing. **Supplementary Table S2.** 16S rRNA gene primers used for quantitative PCR. **Supplementary Table S3.** qPCR primers for selected resistance genes. **Supplementary Table S4.** Selected numerical metadata from the HI-SEAS IV mission. **Supplementary Figure S1.** The HI-SEAS habitat and its surroundings. **Supplementary Figure S2.** Scheme of the HI-SEAS habitat, with sampling locations. The “Sea Can” annexed to the dome contained most food stocks and a workshop. Before exit from, and reentry into, the habitat for extravehicular activities, the crew members remained 5 minutes inside the airlock, with all doors closed. The area adjacent to the telemetry room was similarly used as an airlock, on rare occasions, for bringing large objects into or out of the habitat (door not represented here). The outside door and the door joining the airlock to the dome were never opened at the same time. KC: kitchen floor; MR: desk in the main room; TB: toilet bowl; BR: desk in bedroom. **Supplementary Figure S3.** Boxplot of sample richness (observed ASVs – amplicon sequence variants) of individual crew members and built environment locations of the HI-SEAS habitat. **Supplementary Figure S4.** Shannon diversity (H’) according to different sampling material. **Supplementary Figure S5.** Confusion matrix based on supervised learning methods (RandomForest classification) of predicted sample origins. Abbreviations for sampling locations inside the HI-SEAS habitat were: BR (bedroom), KC (kitchen floor), MR (main room), and TB (toilet bowl). **Supplementary Figure S6.** Linear mixed effect model of Shannon diversity in response of time and sampling environment (crew skin samples and built environment locations of the HI-SEAS habitat). **Supplementary Figure S7.** Volatility plot of species richness (observed ASVs – amplicon sequencing variants) in the built environment of the HI-SEAS habitat and its crew. **Supplementary Figure S8.** Volatility plot of Pielou’s evenness in the built environment of the HI-SEAS habitat and its crew. **Supplementary Figure S9.** Proportion plot of differential feature abundances using balances in gneiss on genus level for the post isolation control time point (days 336 – 400). The proportion plot shows taxa of the crew and the built environment, which could be responsible to explain the differences between the earlier and the later sampling event in each phase (green and orange bars). Differential numerator taxa are grouped to the top (background color in light blue) and differential denominator taxa are grouped to the bottom (background color in dark blue). **Supplementary Figure S10.** Volatility analysis based on linear regression models with time of *Acinetobacter* from different crew members and sampling locations within the HI-SEAS habitat. **Supplementary Figure S11.** Volatility analysis based on linear regression models with time of *Staphylococcus* from different crew members and sampling locations within the HI-SEAS habitat. **Supplementary Figure S12.** Volatility analysis based on linear regression models with time of *Brevundimonas* resolved to different sampling environments (built environment and crew) as well as individual crew members and sampling locations within the HI-SEAS habitat. **Supplementary Figure S13.** Volatility analysis based on linear regression models with time of *Kocuria* resolved to different sampling environments (built environment and crew) as well as individual crew members and sampling locations within the HI-SEAS habitat. **Supplementary Figure S14.** Volatility analysis based on linear regression models with time of *Propionibacterium* resolved to different sampling environments (built environment and crew) as well as individual crew members and sampling locations within the HI-SEAS habitat. **Supplementary Figure S15.** Volatility analysis based on linear regression models with time of *Streptococcus* resolved to different sampling environments (built environment and crew) as well as individual crew members and sampling locations within the HI-SEAS habitat. **Supplementary Figure S16.** Volatility analysis based on linear regression models with time of *Kytococcus* resolved to different sampling environments (built environment and crew) as well as individual crew members and sampling locations within the HI-SEAS habitat. **Supplementary Figure S17.** Volatility analysis based on linear regression models with time of Dermacoccaceae resolved to different sampling environments (built environment and crew) as well as individual crew members and sampling locations within the HI-SEAS habitat. **Supplementary Figure S18.** Volatility analysis based on linear regression models with time of *Gardnerella* resolved to different sampling environments (built environment and crew) as well as individual crew members and sampling locations within the HI-SEAS habitat. **Supplementary Figure S19.** Volatility analysis based on linear regression models with time of *Lactococcus* resolved to different sampling environments (built environment and crew) as well as individual crew members and sampling locations within the HI-SEAS habitat. **Supplementary Figure S20.** Volatility analysis based on linear regression models with time of *Faecalibacterium* resolved to different sampling environments (built environment and crew) as well as individual crew members and sampling locations within the HI-SEAS habitat. **Supplementary Figure S21.** Volatility analysis based on linear regression models with time of *Methanobrevibacter sp.* from different crew members and sampling locations within the HI-SEAS habitat. **Supplementary Figure S22.** Volatility analysis based on linear regression models with time of Archaea and Bacteria from the crew and the HI-SEAS habitat. **Supplementary Figure S23.** Volatility analysis based on linear regression models with time of Enterobacteriaceae resolved to different sampling environments (built environment and crew) as well as individual crew members and sampling locations within the HI-SEAS habitat. **Supplementary Figure S24.** Volatility analysis based on linear regression models with time of *Enhydrobacter* resolved to different sampling environments (built environment and crew) as well as individual crew members and sampling locations within the HI-SEAS habitat. **Supplementary Figure S25.** Volatility analysis based on linear regression models with time of *Pseudomonas* resolved to different sampling environments (built environment and crew) as well as individual crew members and sampling locations within the HI-SEAS habitat. **Supplementary Figure S26.** Volatility analysis of archaeal 16S rRNA gene copies per m^2^ according to qPCR.

## Data Availability

All the raw and processed data used for analyses in this study have been deposited in QIITA under the study ID 12858 (https://qiita.ucsd.edu/; Gonzalez et al. 2018) and EBI-ENA under the accession number ERP118380.

## Abbreviations

ANCOM: Analysis of composition of microbiomes
ASVs: Amplicon sequence variants
BHP: Behavioral Health and Performance
CSA: Canadian Space Agency
FDR: False discovery rate
HI-SEAS: Hawaii Space Exploration Analog and Simulation mission
ICE: Isolated and confined built environments
ILMAH: Inflatable lunar/Mars analogous habitat
ISS: International Space Station
JAXA: Japan Aerospace Exploration Agency
LEfSe: Linear discriminant analysis Effect Size
MoBE: Microbiome of the built environment
NASA: National Aeronautics and Space Administration
PCR: Polymerase chain reaction
qPCR: Quantitative polymerase chain reaction
PERMANOVA: Permutational multivariate analysis of variance
QIIME: Quantitative Insights Into Microbial Ecology
RDA: Redundancy analysis
SD: Standard deviation
