# Supplementary material for: Microbiome dynamics during the HI-SEAS IV mission, and implications for future crewed missions beyond Earth
Source: Microbiome. 2021 Jan 24;9:27. doi: 10.1186/s40168-020-00959-x (PMC7831191; doi:10.1186/s40168-020-00959-x)
Supplement: Supplementary file 2 — Additional file 1: Supplementary Table S1. 16S rRNA gene primers used for generating the amplicons for Illumina MiSeq sequencing. Supplementary Table S2. 16S rRNA gene primers used for quantitative PCR. Supplementary Table S3. qPCR primers for selected resistance genes. Supplementary Table S4. Selected numerical metadata from the HI-SEAS IV mission. Supplementary Figure S1. The HI-SEAS habitat and its surroundings. Supplementary Figure S2. Scheme of the HI-SEAS habitat, with sampling locations. The “Sea Can” annexed to the dome contained most food stocks and a workshop. Before exit from, and reentry into, the habitat for extravehicular activities, the crew members remained 5 minutes inside the airlock, with all doors closed. The area adjacent to the telemetry room was similarly used as an airlock, on rare occasions, for bringing large objects into or out of the habitat (door not represented here). The outside door and the door joining the airlock to the dome were never opened at the same time. KC: kitchen floor; MR: desk in the main room; TB: toilet bowl; BR: desk in bedroom. Supplementary Figure S3. Boxplot of sample richness (observed ASVs – amplicon sequence variants) of individual crew members and built environment locations of the HI-SEAS habitat. Supplementary Figure S4. Shannon diversity (H’) according to different sampling material. Supplementary Figure S5. Confusion matrix based on supervised learning methods (RandomForest classification) of predicted sample origins. Abbreviations for sampling locations inside the HI-SEAS habitat were: BR (bedroom), KC (kitchen floor), MR (main room), and TB (toilet bowl). Supplementary Figure S6. Linear mixed effect model of Shannon diversity in response of time and sampling environment (crew skin samples and built environment locations of the HI-SEAS habitat). Supplementary Figure S7. Volatility plot of species richness (observed ASVs – amplicon sequencing variants) in the built environment of the HI-SEAS habitat and its [file 40168_2020_959_MOESM2_ESM.pdf]

Supplementary

Title: Microbiome dynamics during the HI-SEAS IV mission, and implications for future crewed missions beyond Earth

Authors: Alexander Mahnert[1], Cyprien Verseux[2], Petra Schwendner[3], Kaisa Koskinen[1,4], Christina Kumpitsch[1], Marcus Blohs[1], Lisa Wink[1], Daniela Brunner[1], Theodora Goessler[1], Daniela Billi[5] and Christine Moissl-Eichinger[1,4*]

[1]Interactive Microbiome Research, Diagnostic & Research Institute of Hygiene, Microbiology and Environmental Medicine, Medical University of Graz, Neue Stiftingtalstrasse 6, 8010 Graz, Austria

[2]Laboratory of Applied Space Microbiology, Center of Applied Space Technology and Microgravity (ZARM), University of Bremen, Am Fallturm 2, 28359 Bremen, Germany

[3]University of Florida, Space Life Sciences Lab, 505 Odyssey Way, Exploration Park, N. Merritt Island, FL 32953, USA

[4]BioTechMed-Graz

[5]Department of Biology, University of Rome Tor Vergata, Via della Ricerca Scientifica s.n.c, 00133 Rome, Italy

* corresponding author: christine.moissl-eichinger@medunigraz.at

Author contact details: AM (alexander.mahnert@medunigraz.at), CV (cyprien.verseux@gmail.com), PS (petra.schwendner@ufl.edu), KK, (kaisa.koskinen@medunigraz.at), CK (christina.kumpitsch@medunigraz.at), MB (marcus.blohs@medunigraz.at), LW (lisa.wink@medunigraz.at), DBr (danbrun0103@gmail.com), TG (theodora.kopun@gmail.com), DBi (billi@uniroma2.it), CM-E (christine.moissl-eichinger@medunigraz.at)

Supplementary Tables

Supplementary Table S1: 16S rRNA gene primers used for generating the amplicons for Illumina MiSeq sequencing.

16S rRNA gene amplicon primers

primer name	primer sequence	target
Illumina-tagged primers F515	(5'-TCGTCGGCAGCGTCAGATGTGTATAAGAGACAG GTGCCAGCMGCCGCGGTAA-3')	Bacteria and some Archaea
Illumina-tagged primers R806	(5'-GTCTCGTGGGCTCGGAGATGTGTATAAGAGACAG GGACTACHVGGGTWTCTAAT-3')	Bacteria and some Archaea

Supplementary Table S2: 16S rRNA gene primers used for quantitative PCR.

16S rRNA gene primers for qPCR

primer name	primer sequence	target
Bac_331F	(5'-TCCTACGGGAGGCAGCAGT-3')	Bacteria
Bac_797R	(5'-GGACTACCAGGGTATCTAATCCTGTT-3')	Bacteria
A806F	(5'-ATTAGATACCCSBGTAGTCC-3')	Archaea
A958R	(5'-YCCGGCGTTGAMTCCAATT-3')	Archaea

Supplementary Table S3: qPCR primers for selected resistance genes.

qPCR primers for selected resistance genes

primer name	primer sequence	target
int1-a F1	(5'-CGAAGTCGAGGCATTTCTGTC -3')	class 1 integrase
int1-a R1	(5'-GCCTTCCAGAAAACCGAGGA-3')	
qacEΔ1 F1	(5'-TCGCAACATCCGCATTAAAA-3')	biocide resistance gene, quaternary ammonium compound-resistance
qacEΔ1 R1	(5'-GGATTTCAGAACCAGAGAAAGAAA-3')	
tetM F1	(5'-CATCATAGACACGCCAGGACA-3')	tetracycline resistance
tetM R1	(5'-CTGTTTGATTACAATTTCCGC-3')	
blaOXA-58 F1	(5'-GTGCTGAGCATAGTATGA-3')	class A beta-lactamase
blaOXA-58 R1	(5'-CGGTCTAAATGCGTGCCA-3')	

Supplementary Table S4: Selected numerical metadata from the HI-SEAS IV mission

Selected numerical metadata of the study

category	mean	standard deviation	min	max
temperature [°C] in Utility module	28,06	1,34	25,43	30,52
temperature [°C] HI-SEAS habitat downstairs	17,64	1,11	14,45	30,52
temperature [°C] HI-SEAS habitat upstairs	18,88	1,50	15,45	21,46
CO_2 [ppm] HI-SEAS habitat	661,64	61,77	561,87	908,72
crew age [years]	30	4	25	36
crew height [cm]	177	9	170	189
number of showers	61	16	35	79
days in between two shower events	5	2	4	9
duration of showers [seconds]	102	47	40	148

Supplementary Figures

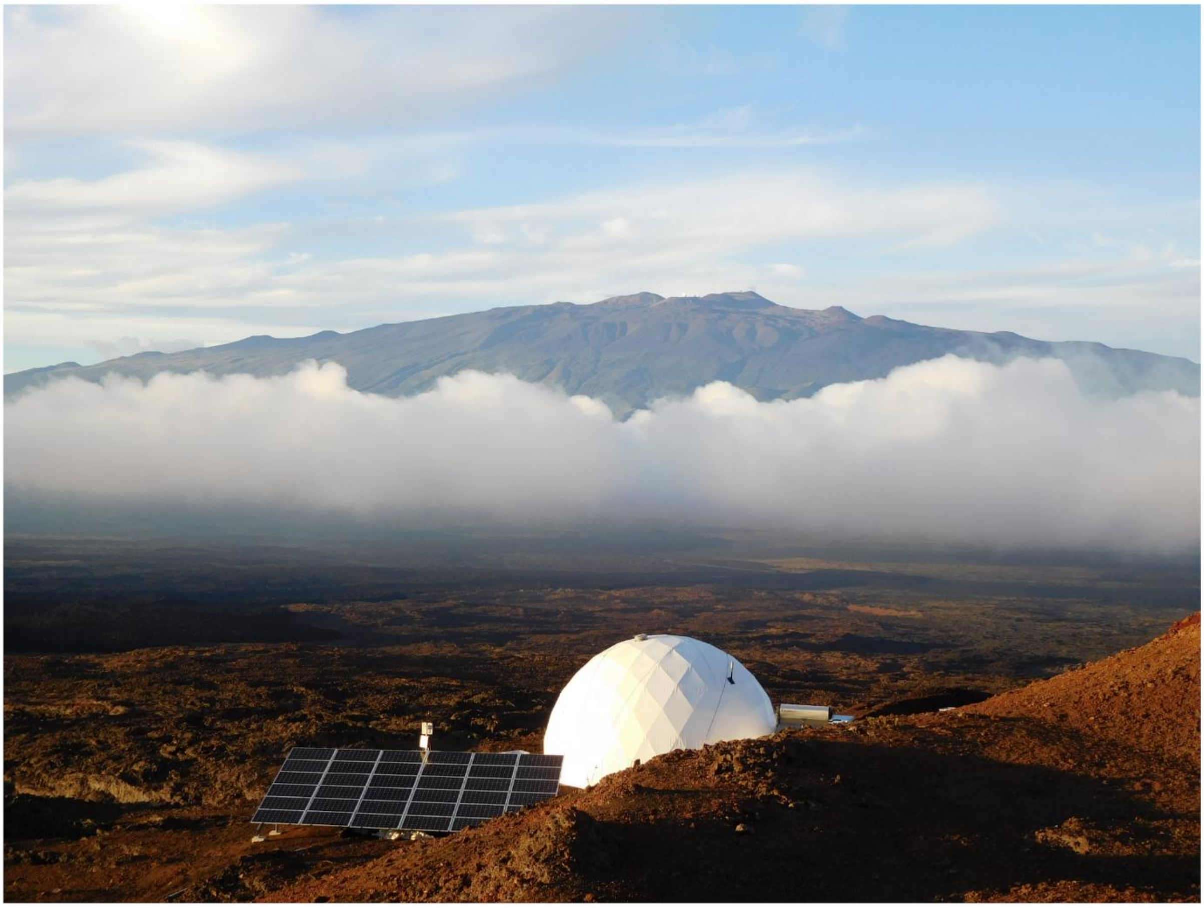

Supplementary Figure S1: The HI-SEAS habitat and its surroundings.

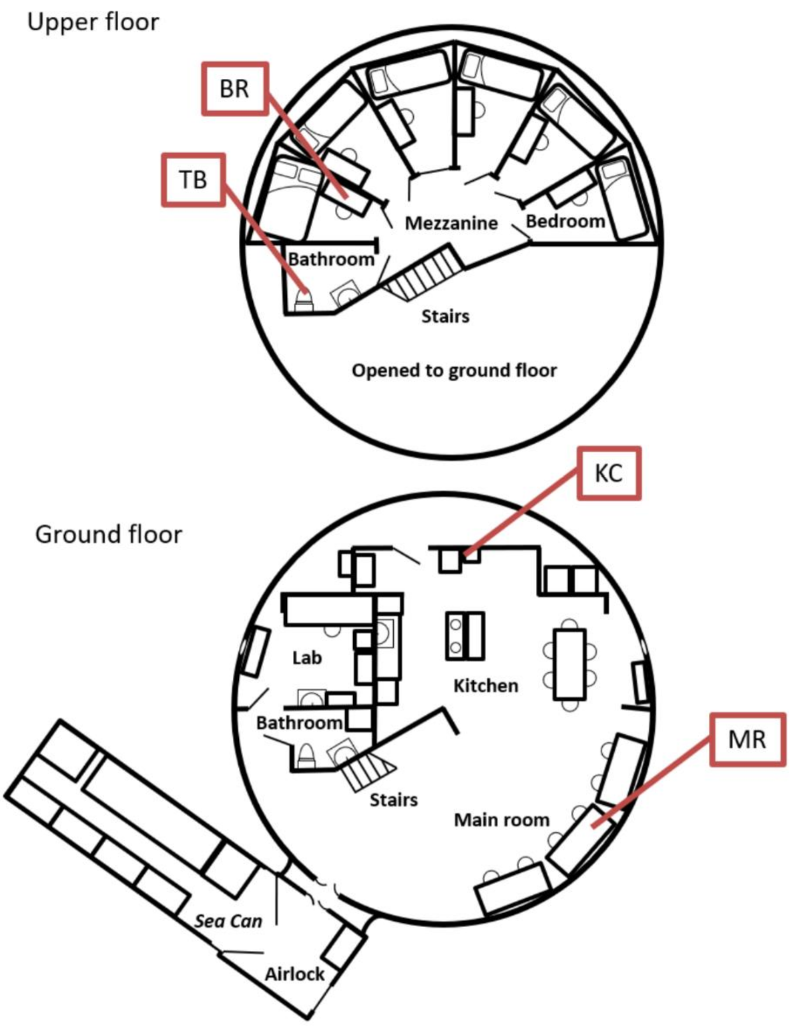

Supplementary Figure S2: Scheme of the HI-SEAS habitat, with sampling locations. The "Sea Can" annexed to the dome contained most food stocks and a workshop. Before exit from, and reentry into, the habitat for extravehicular activities, the crew members remained 5 minutes inside the airlock, with all doors closed. The area adjacent to the telemetry room was similarly used as an airlock, on rare occasions, for bringing large objects into or out of the habitat (door not represented here). The outside door and the door joining the airlock to the dome were never opened at the same time. KC: kitchen floor; MR: desk in the main room; TB: toilet bowl; BR: desk in bedroom.

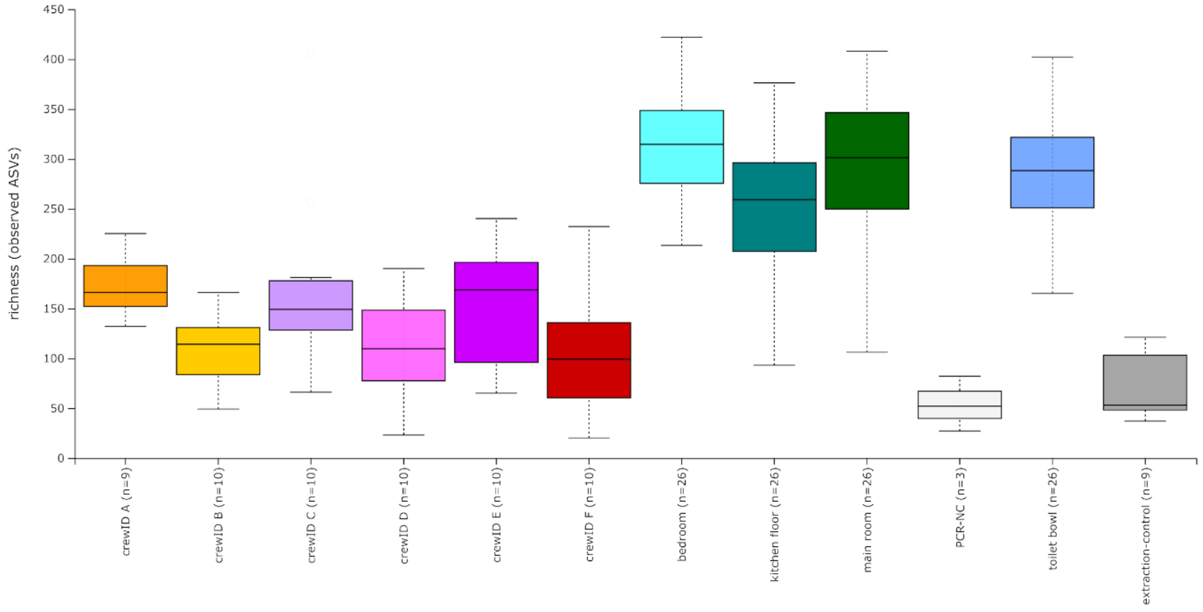

Supplementary Figure S3: Boxplot of sample richness (observed ASVs – amplicon sequence variants) of individual crew members and built environment locations of the HI-SEAS habitat.

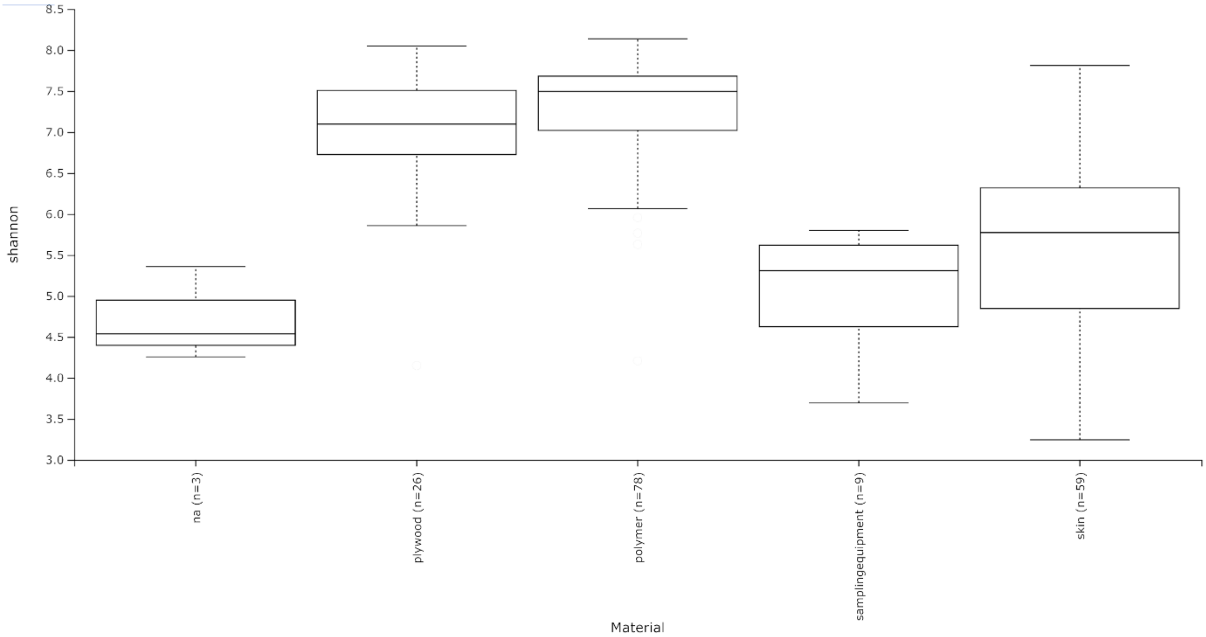

Supplementary Figure S4: Shannon diversity (H’) according to different sampling material.

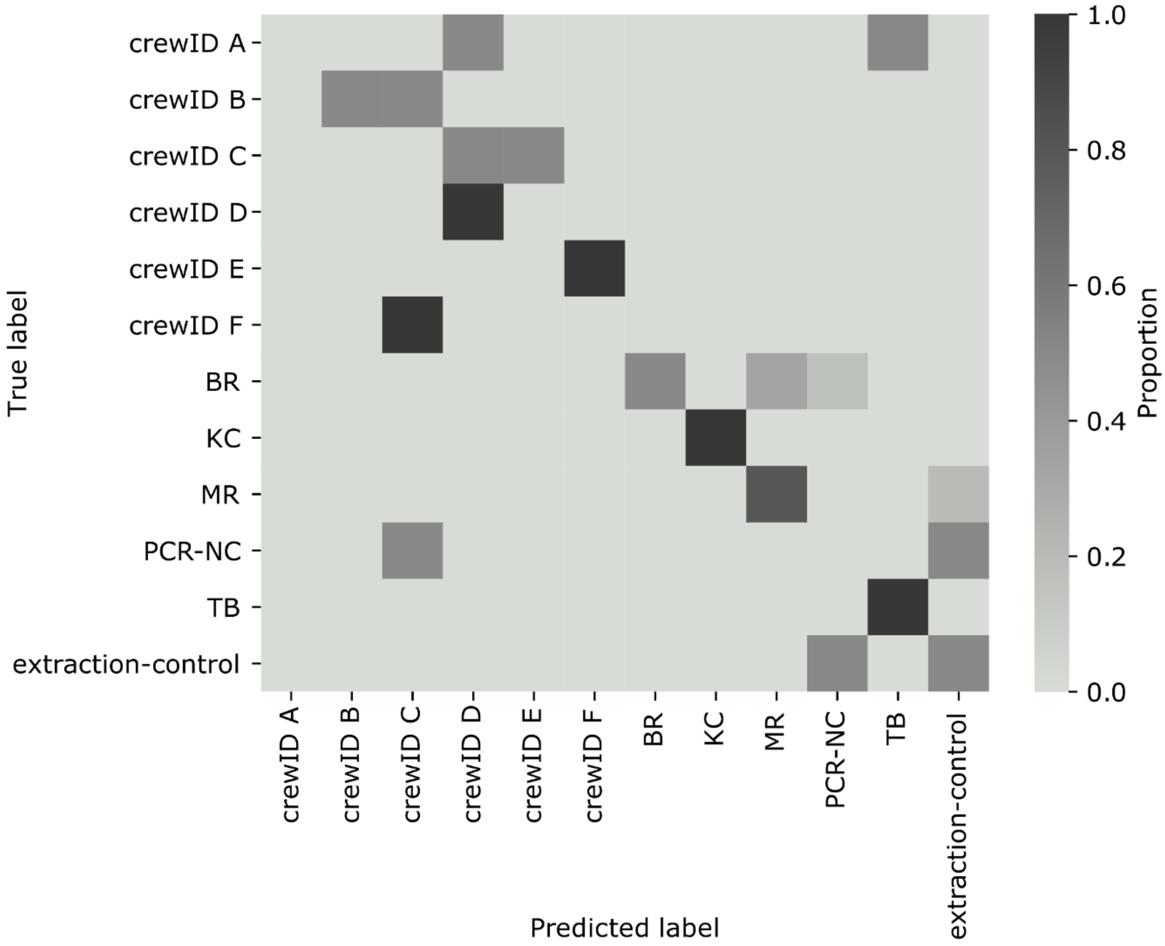

Supplementary Figure S5: Confusion matrix based on supervised learning methods (RandomForest classification) of predicted sample origins. Abbreviations for sampling locations inside the HI-SEAS habitat were: BR (bedroom), KC (kitchen floor), MR (main room), and TB (toilet bowl).

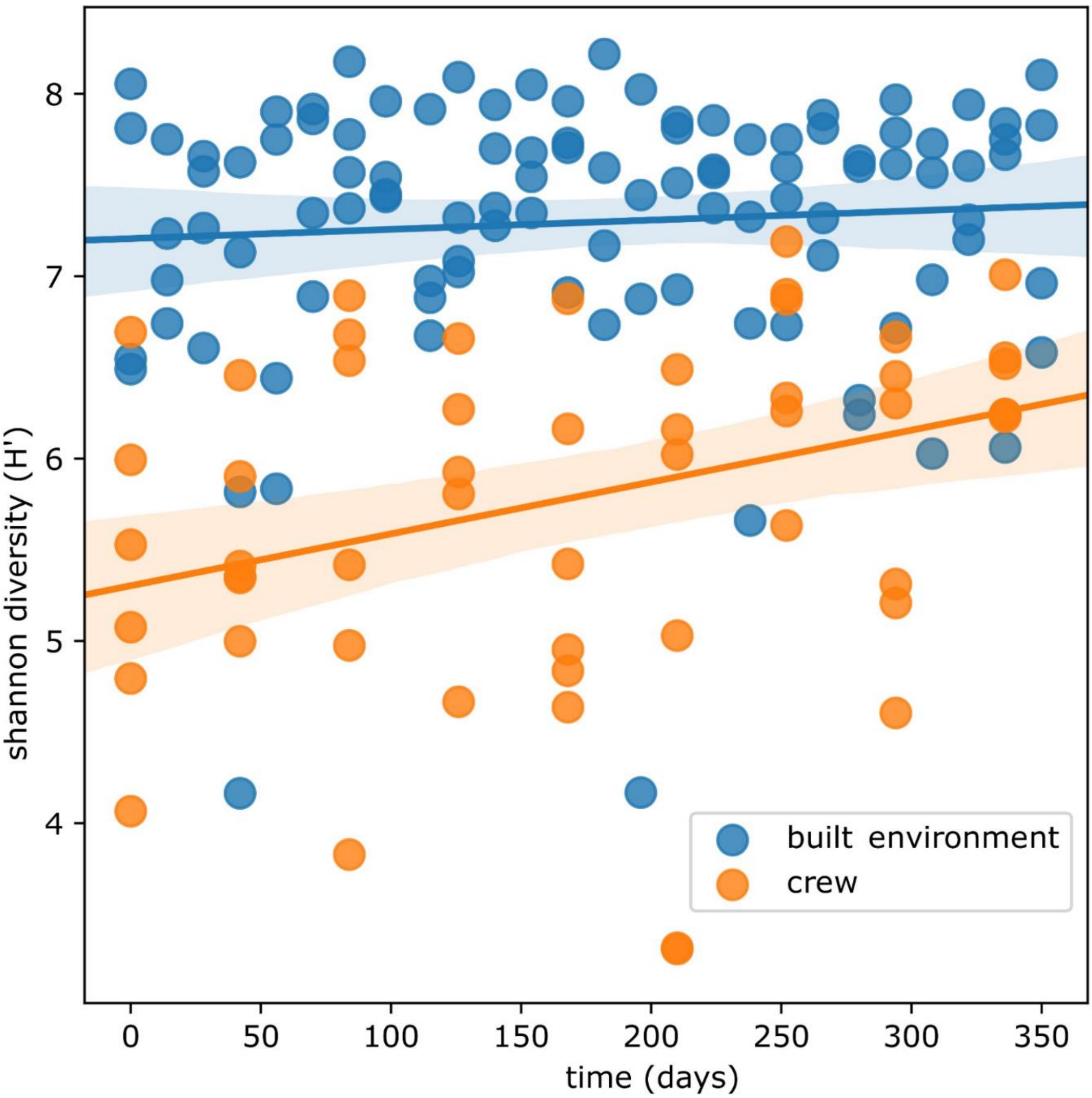

Supplementary Figure S6: Linear mixed effect model of Shannon diversity in response of time and sampling environment (crew skin samples and built environment locations of the HI-SEAS habitat).

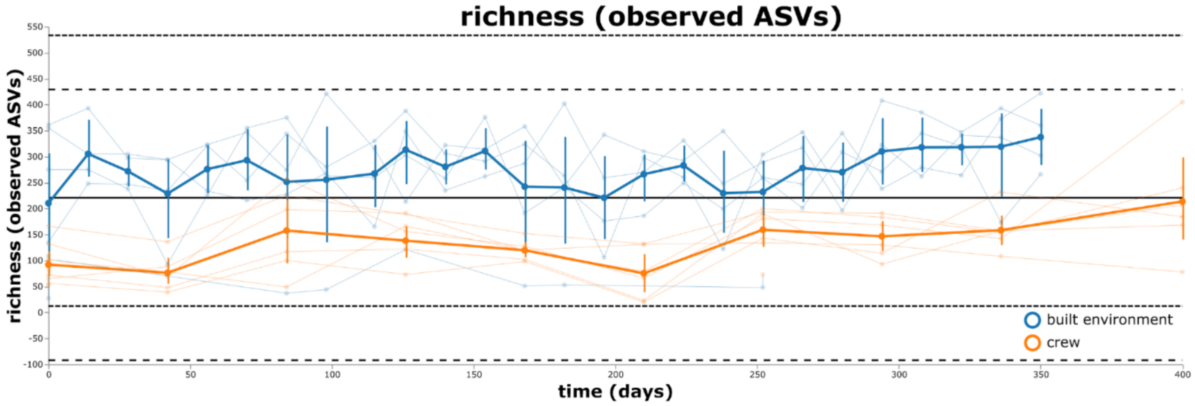

Supplementary Figure S7: Volatility plot of species richness (observed ASVs – amplicon sequencing variants) in the built environment of the HI-SEAS habitat and its crew.

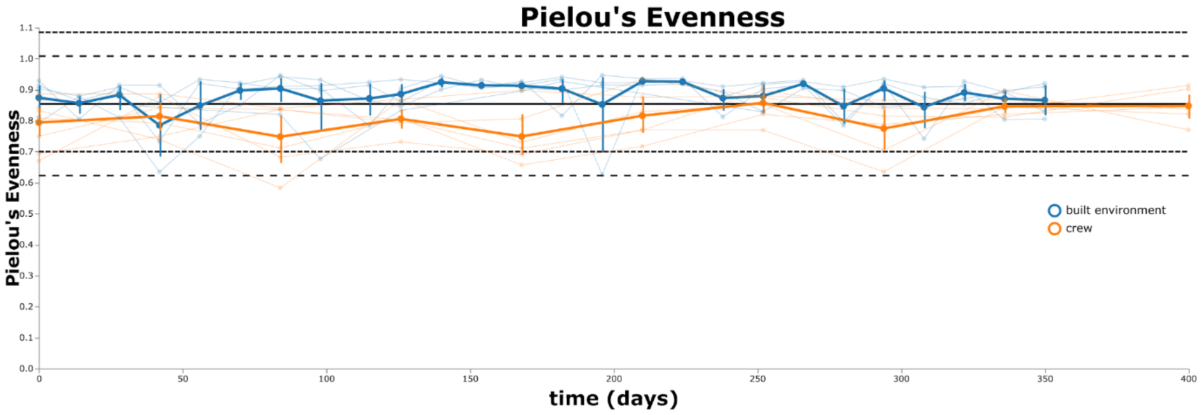

Supplementary Figure S8: Volatility plot of Pielou’s evenness in the built environment of the HI-SEAS habitat and its crew.

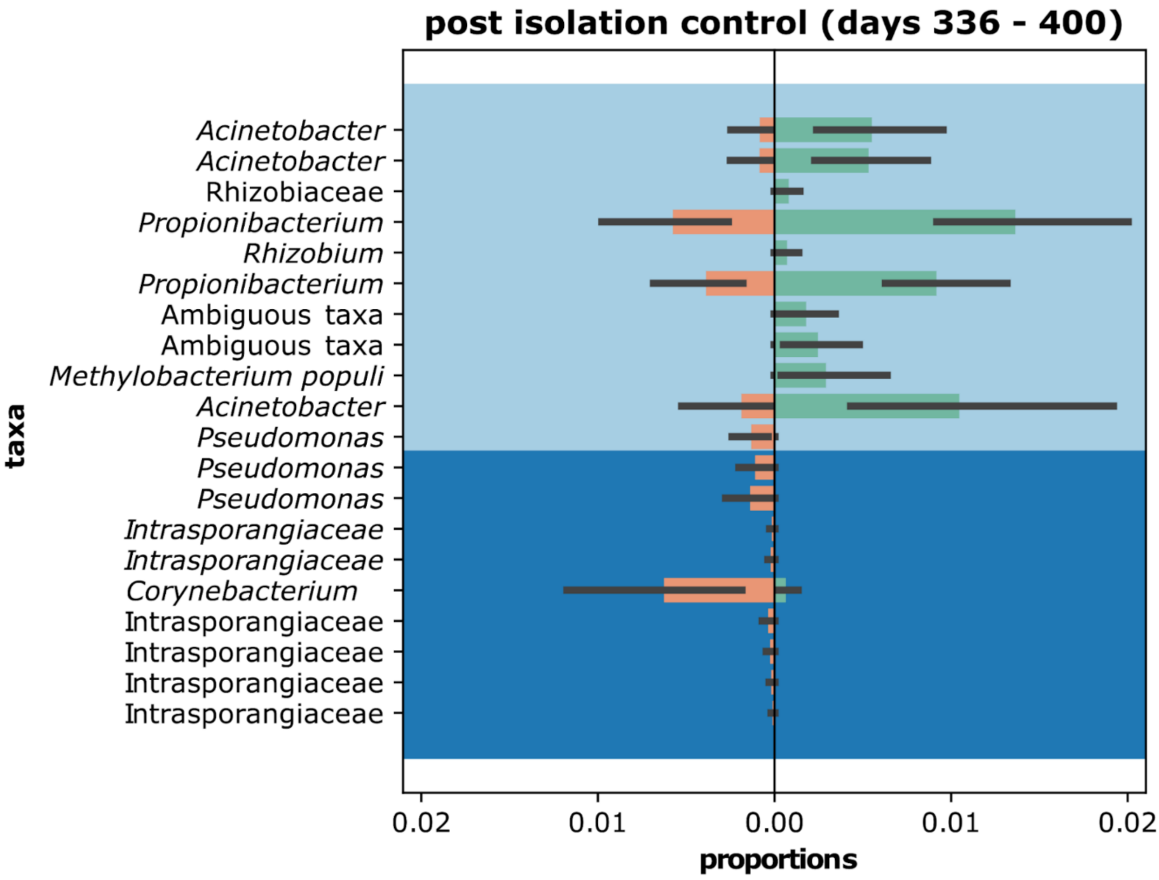

Supplementary Figure S9: Proportion plot of differential feature abundances using balances in gneiss on genus level for the post isolation control time point (days 336 – 400). The proportion plot shows taxa of the crew and the built environment, which could be responsible to explain the differences between the earlier and the later sampling event in each phase (green and orange bars). Differential numerator taxa are grouped to the top (background color in light blue) and differential denominator taxa are grouped to the bottom (background color in dark blue).

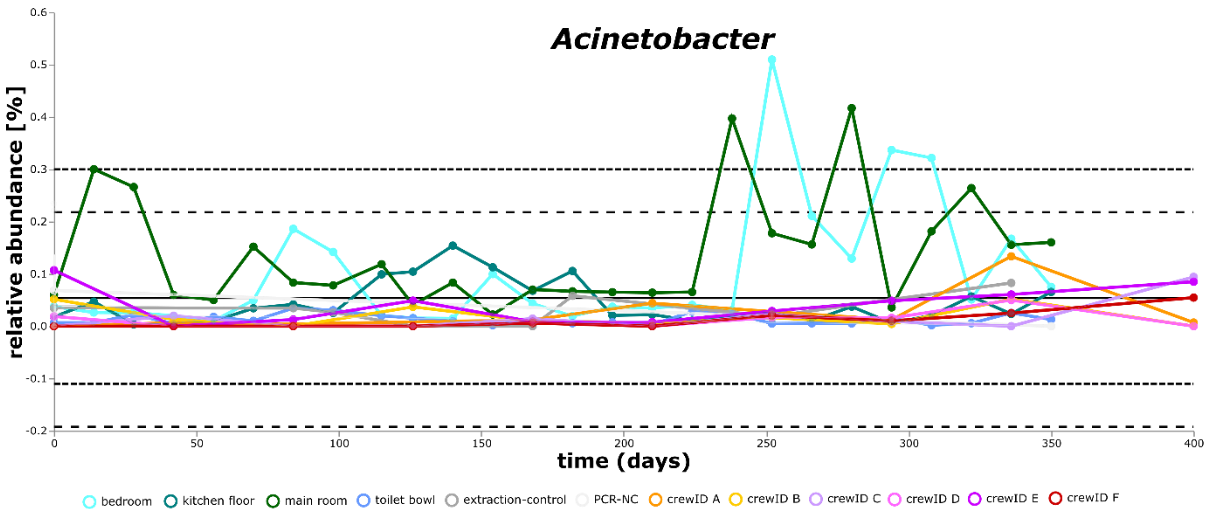

Supplementary Figure S10: Volatility analysis based on linear regression models with time of *Acinetobacter* from different crewmembers and sampling locations within the HI-SEAS habitat.

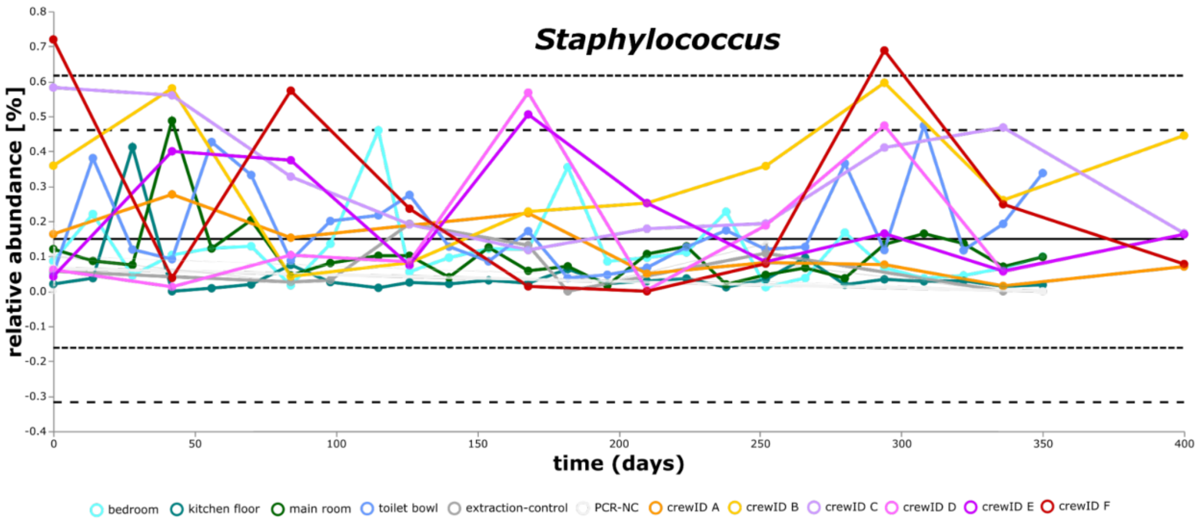

Supplementary Figure S11: Volatility analysis based on linear regression models with time of *Staphylococcus* from different crewmembers and sampling locations within the HI-SEAS habitat.

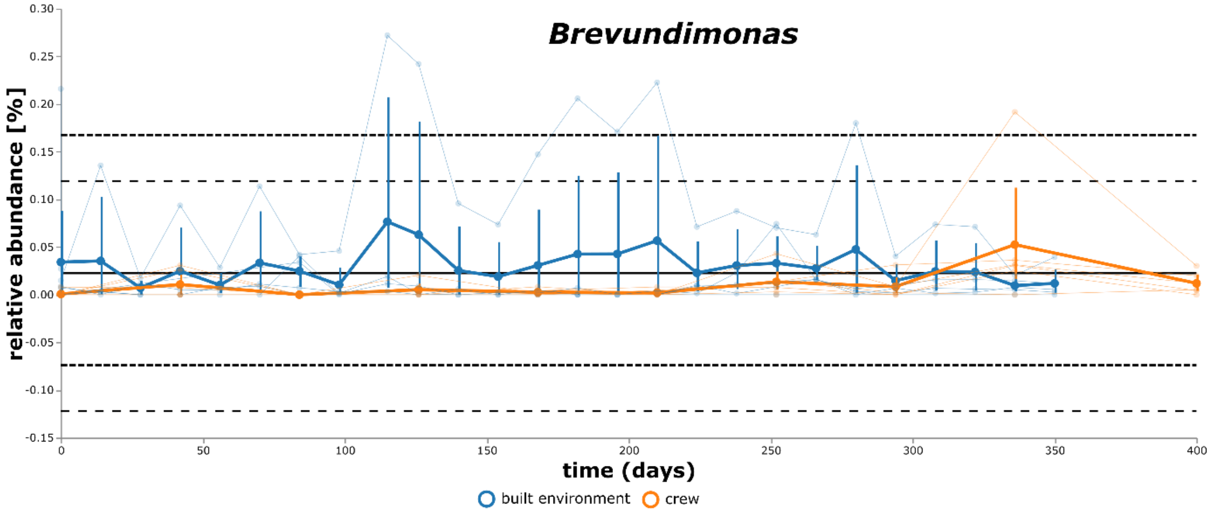

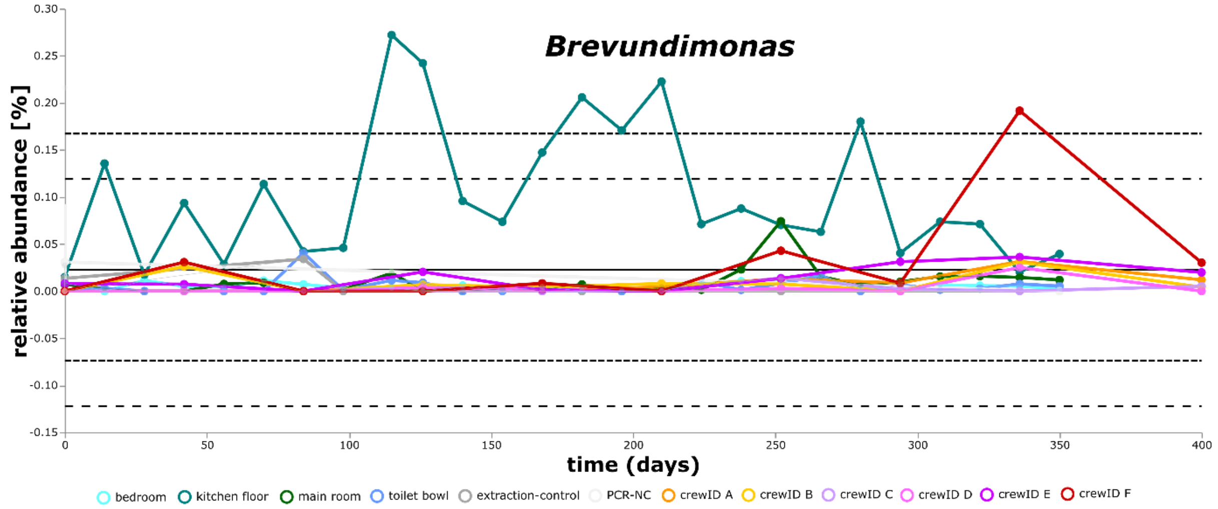

Supplementary Figure S12: Volatility analysis based on linear regression models with time of *Brevundimonas* resolved to different sampling environments (built environment and crew) as well as individual crew members and sampling locations within the HI-SEAS habitat.

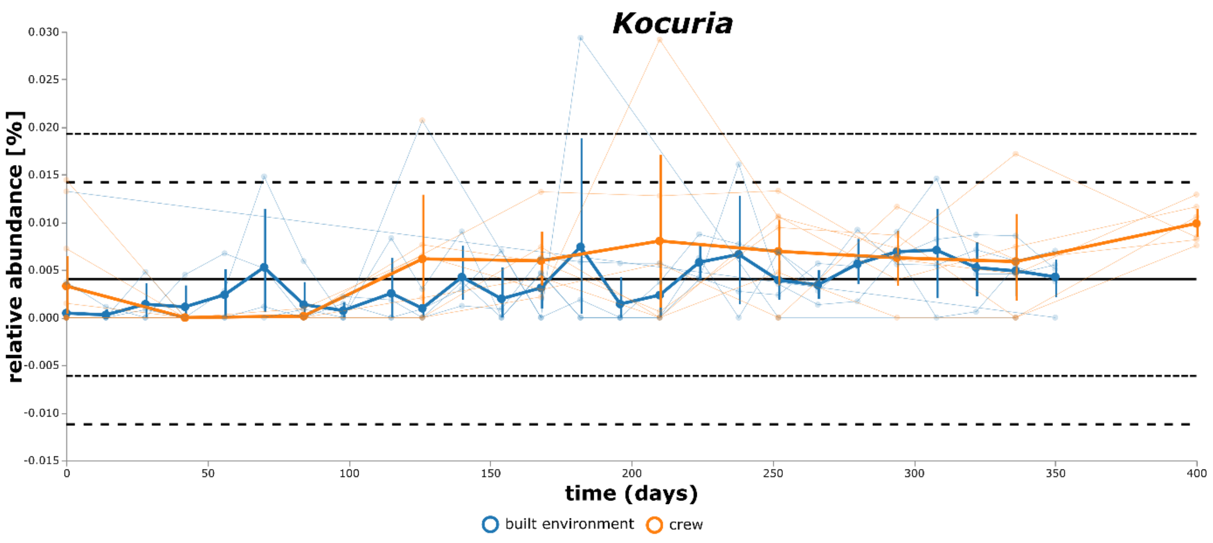

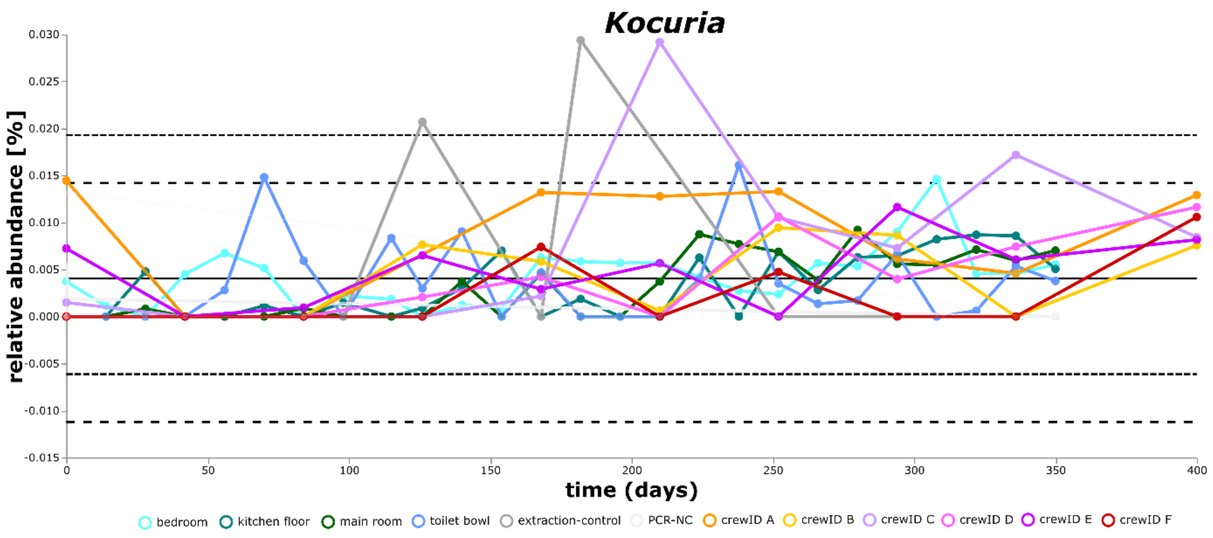

Supplementary Figure S13: Volatility analysis based on linear regression models with time of *Kocuria* resolved to different sampling environments (built environment and crew) as well as individual crew members and sampling locations within the HI-SEAS habitat.

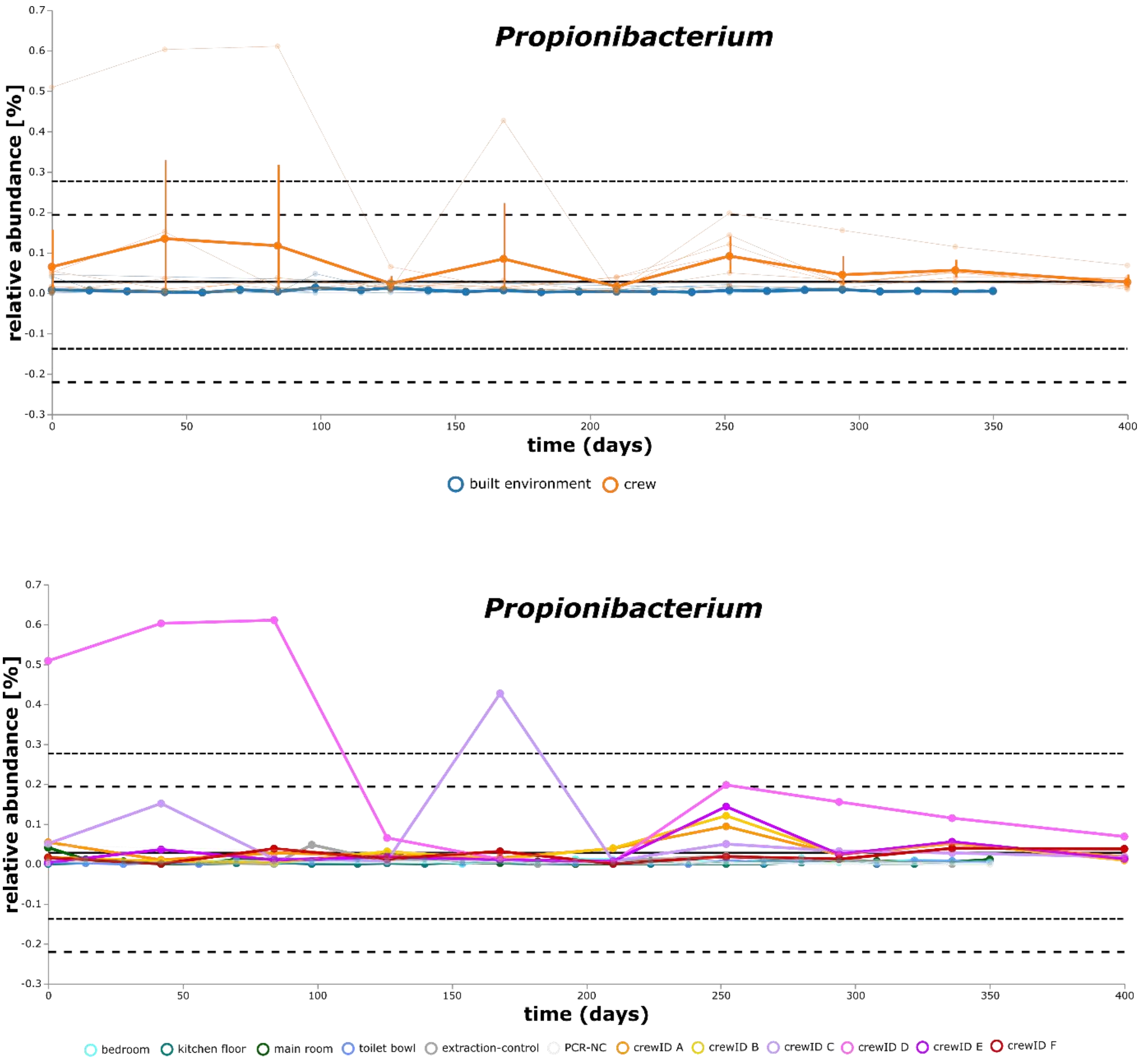

Supplementary Figure S14: Volatility analysis based on linear regression models with time of *Propionibacterium* resolved to different sampling environments (built environment and crew) as well as individual crew members and sampling locations within the HI-SEAS habitat.

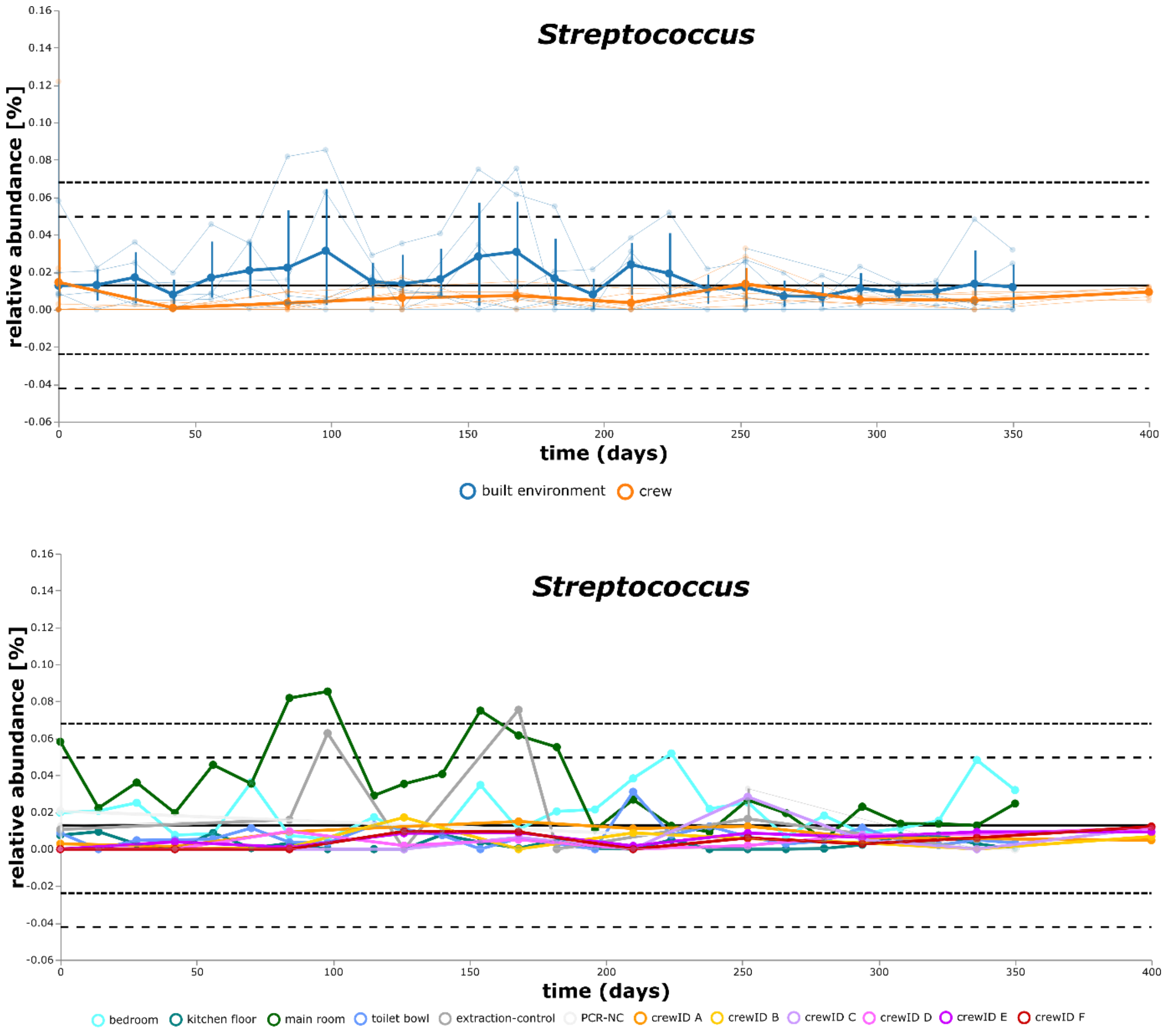

Supplementary Figure S15: Volatility analysis based on linear regression models with time of *Streptococcus* resolved to different sampling environments (built environment and crew) as well as individual crew members and sampling locations within the HI-SEAS habitat.

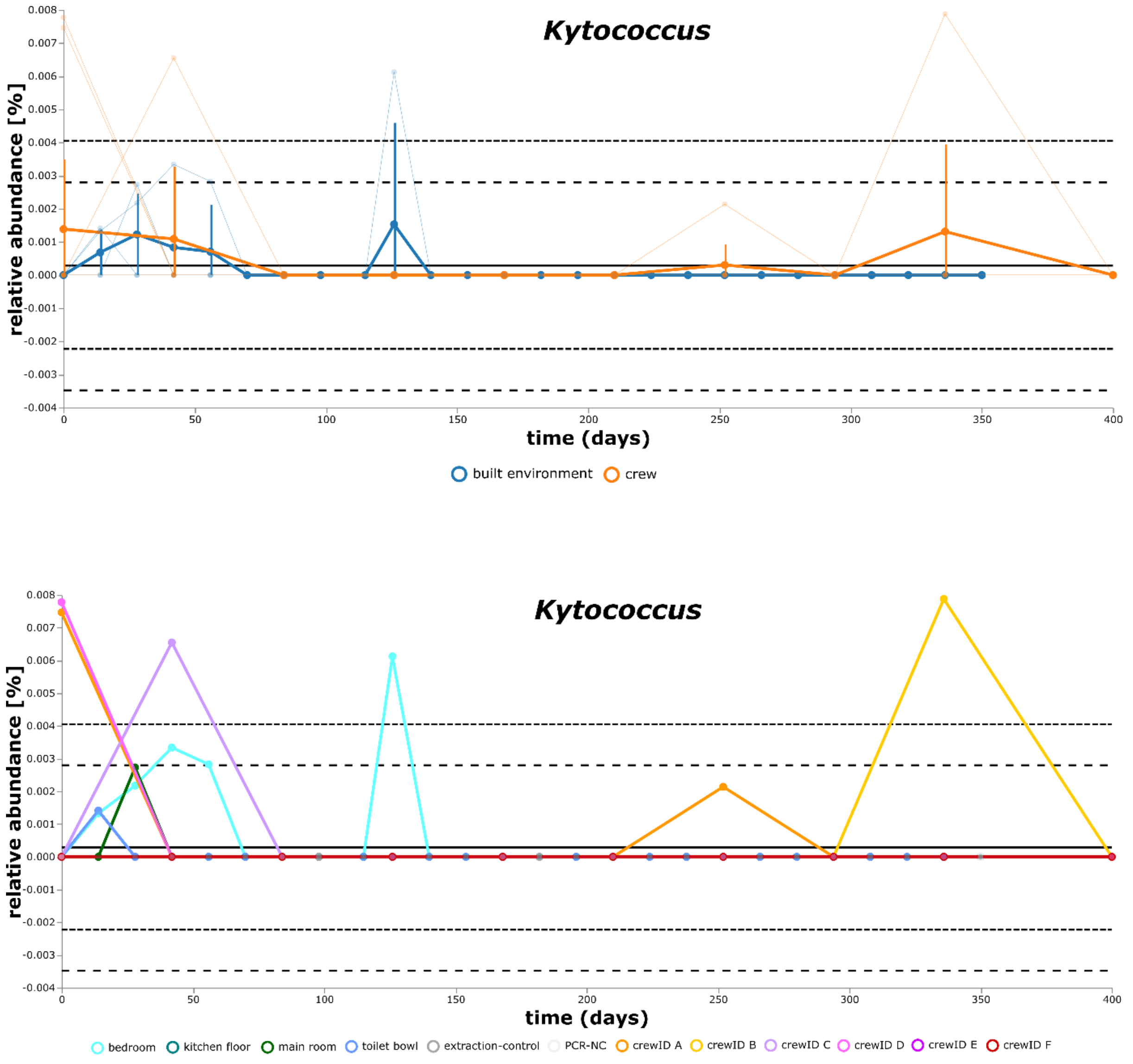

Supplementary Figure S16: Volatility analysis based on linear regression models with time of *Kytococcus* resolved to different sampling environments (built environment and crew) as well as individual crew members and sampling locations within the HI-SEAS habitat.

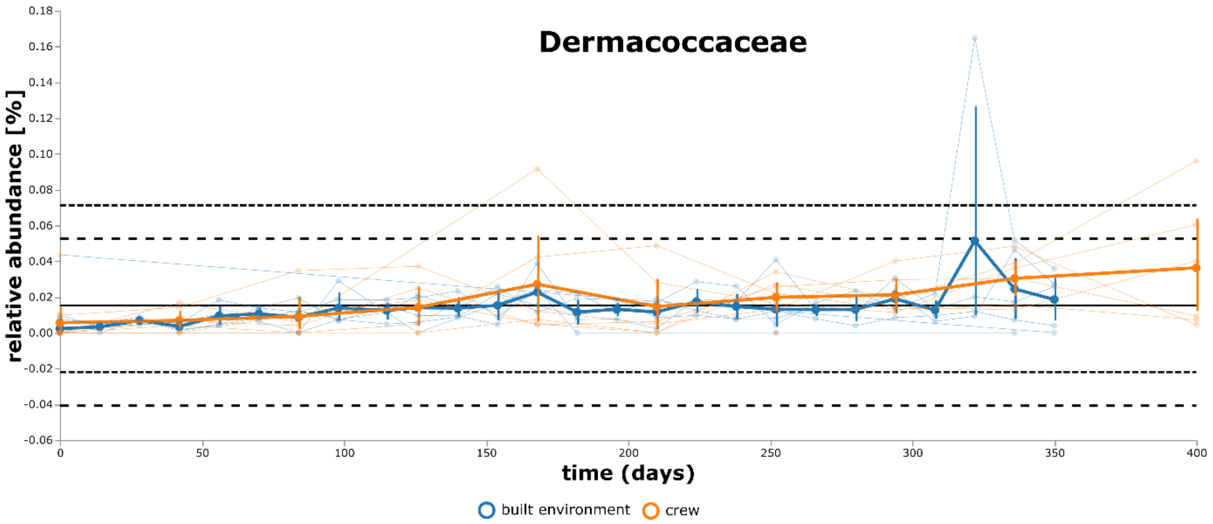

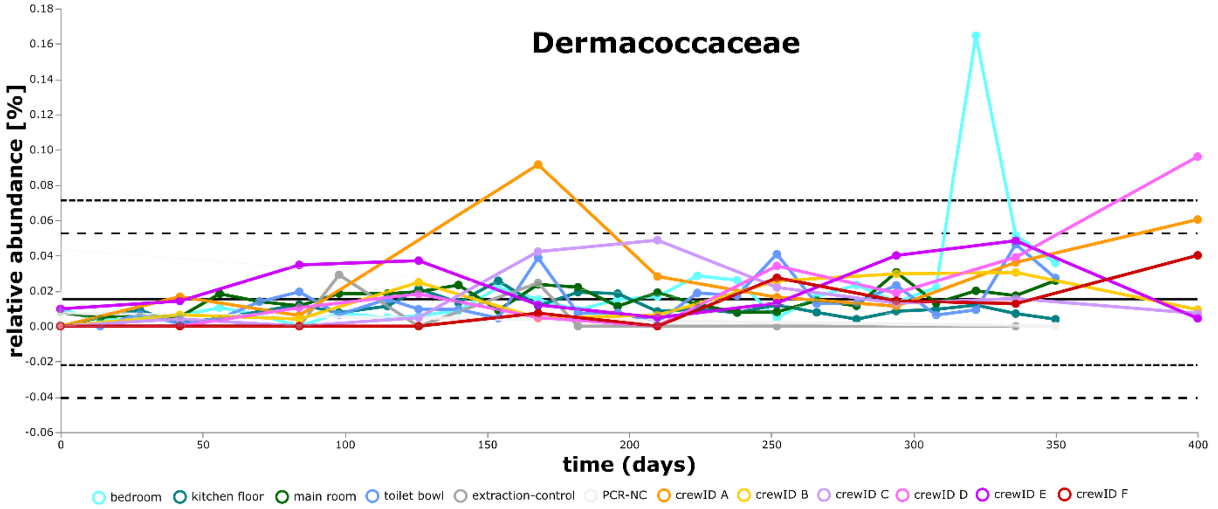

Supplementary Figure S17: Volatility analysis based on linear regression models with time of Dermacoccaceae resolved to different sampling environments (built environment and crew) as well as individual crew members and sampling locations within the HI-SEAS habitat.

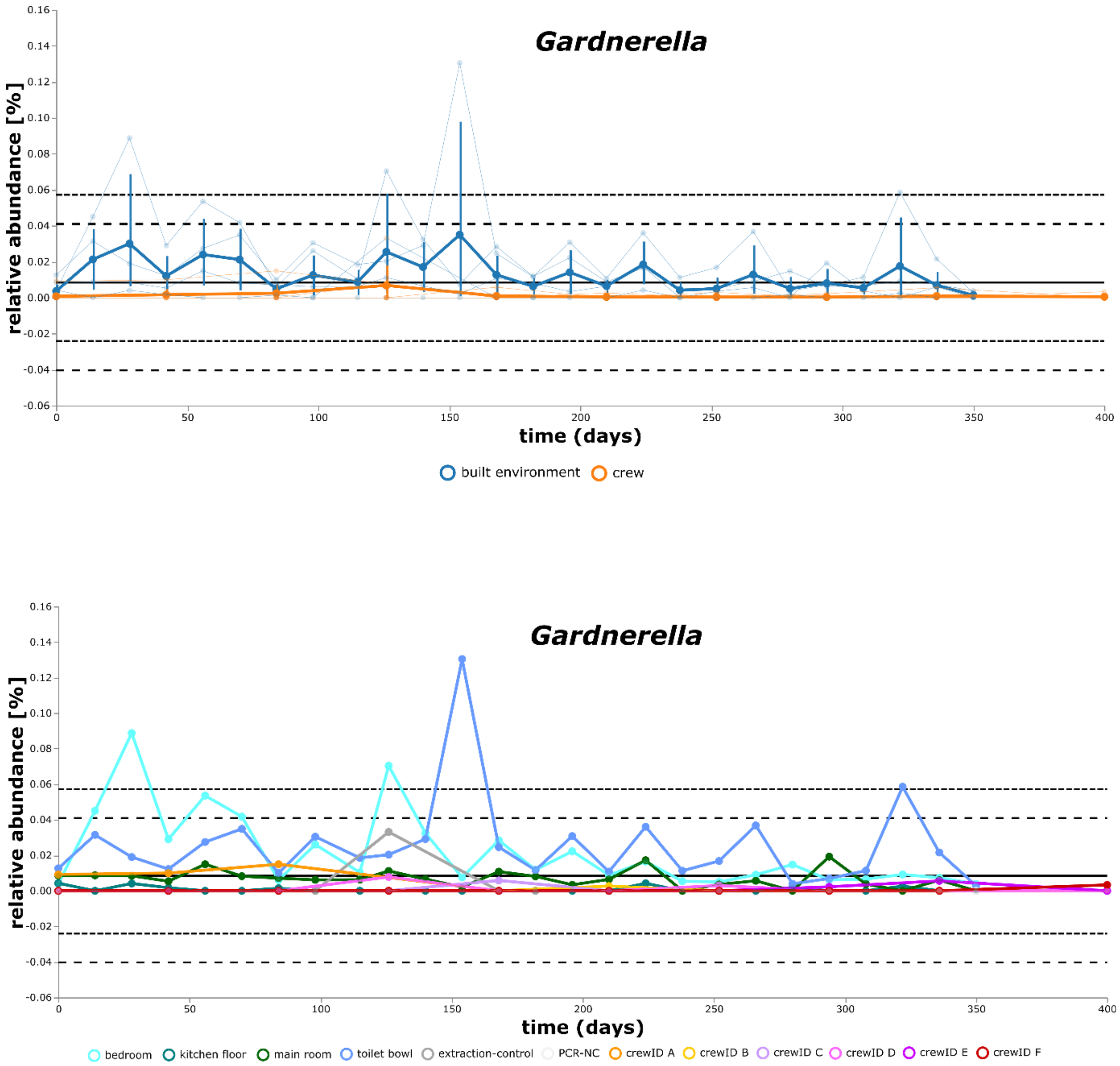

Supplementary Figure S18: Volatility analysis based on linear regression models with time of *Gardnerella* resolved to different sampling environments (built environment and crew) as well as individual crew members and sampling locations within the HI-SEAS habitat.

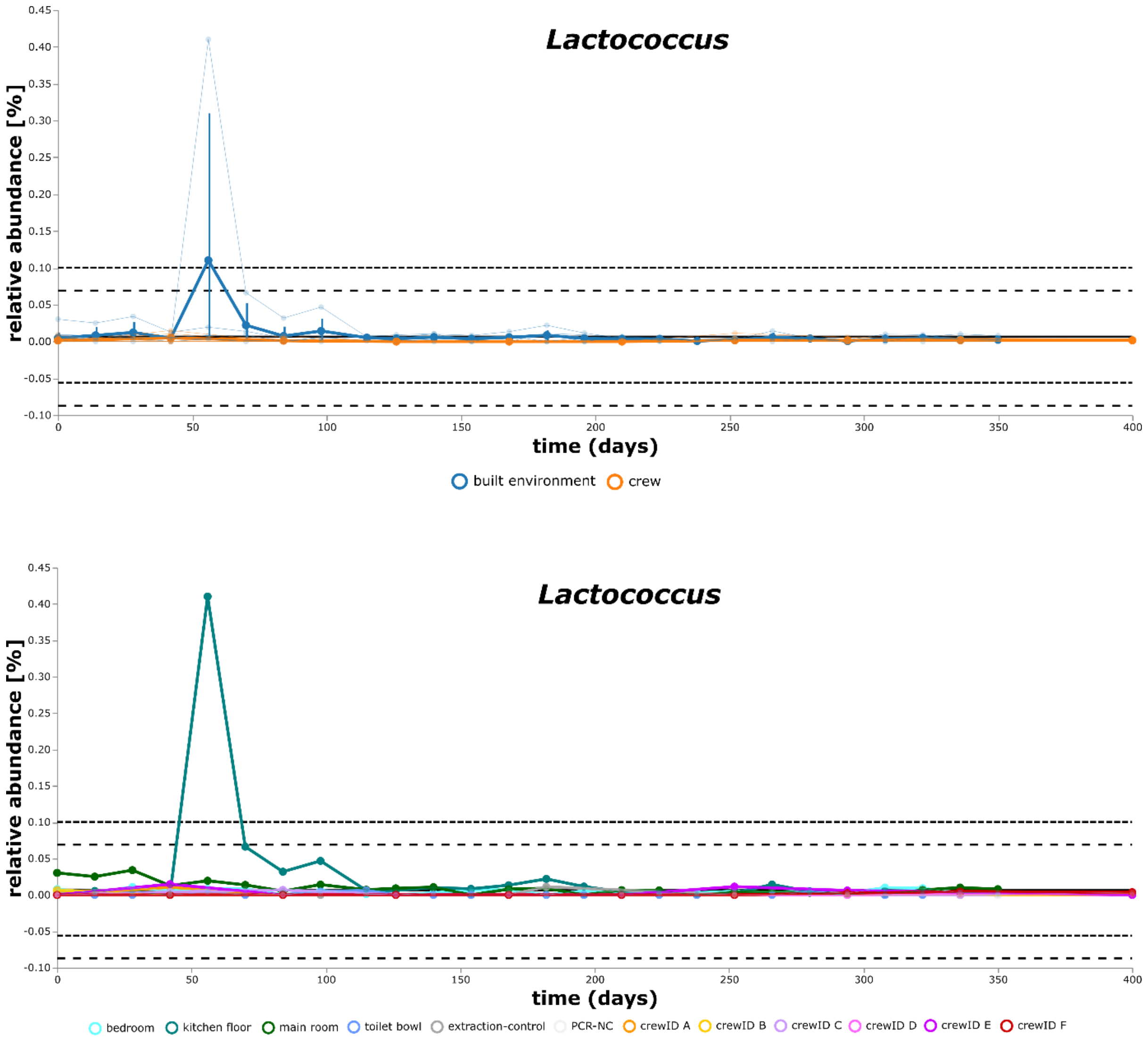

Supplementary Figure S19: Volatility analysis based on linear regression models with time of *Lactococcus* resolved to different sampling environments (built environment and crew) as well as individual crew members and sampling locations within the HI-SEAS habitat.

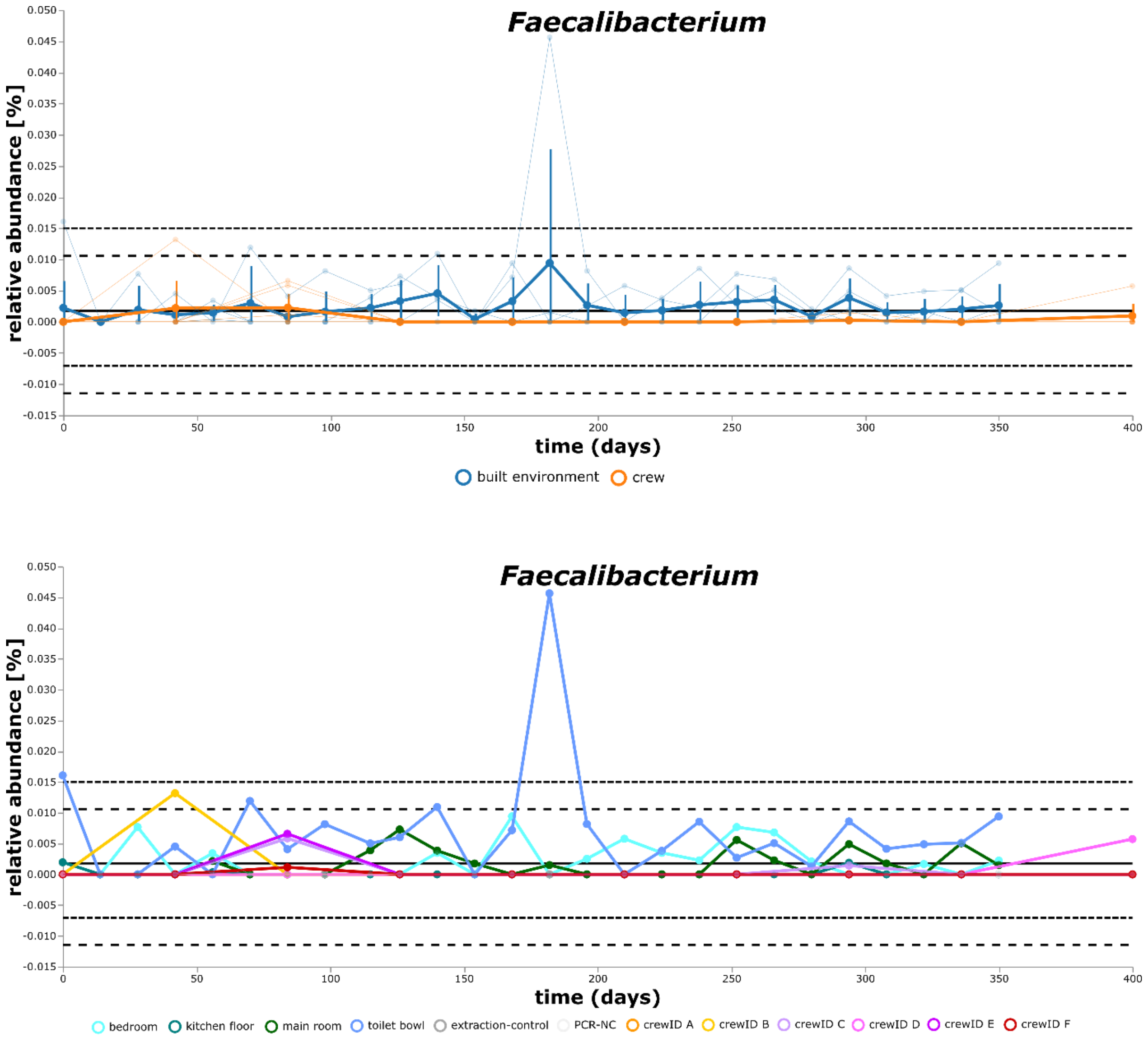

Supplementary Figure S20: Volatility analysis based on linear regression models with time of *Faecalibacterium* resolved to different sampling environments (built environment and crew) as well as individual crew members and sampling locations within the HI-SEAS habitat.

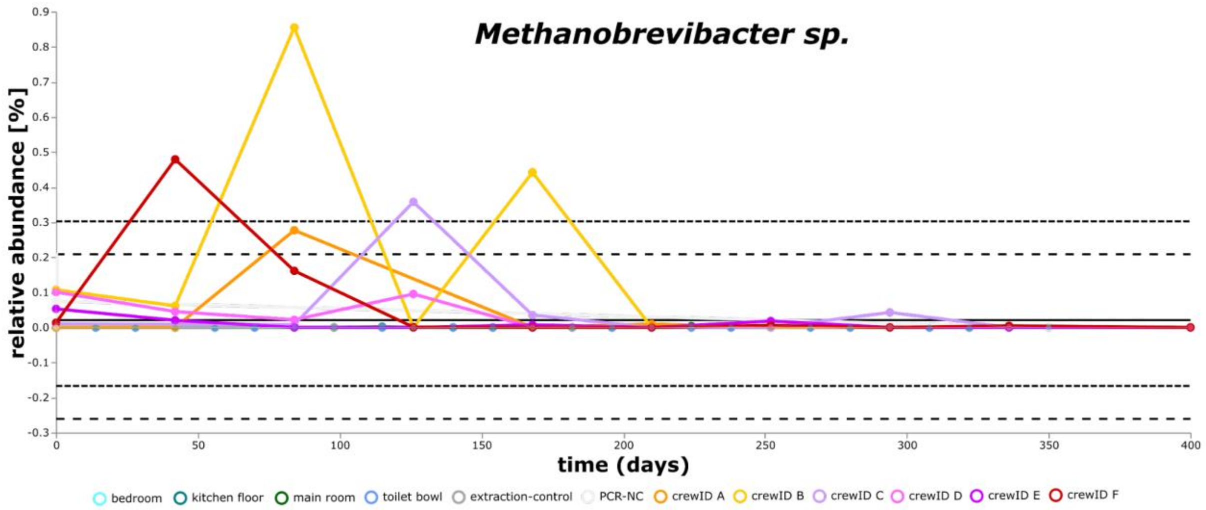

Supplementary Figure S21: Volatility analysis based on linear regression models with time of *Methanobrevibacter sp.* from different crew members and sampling locations within the HI-SEAS habitat.

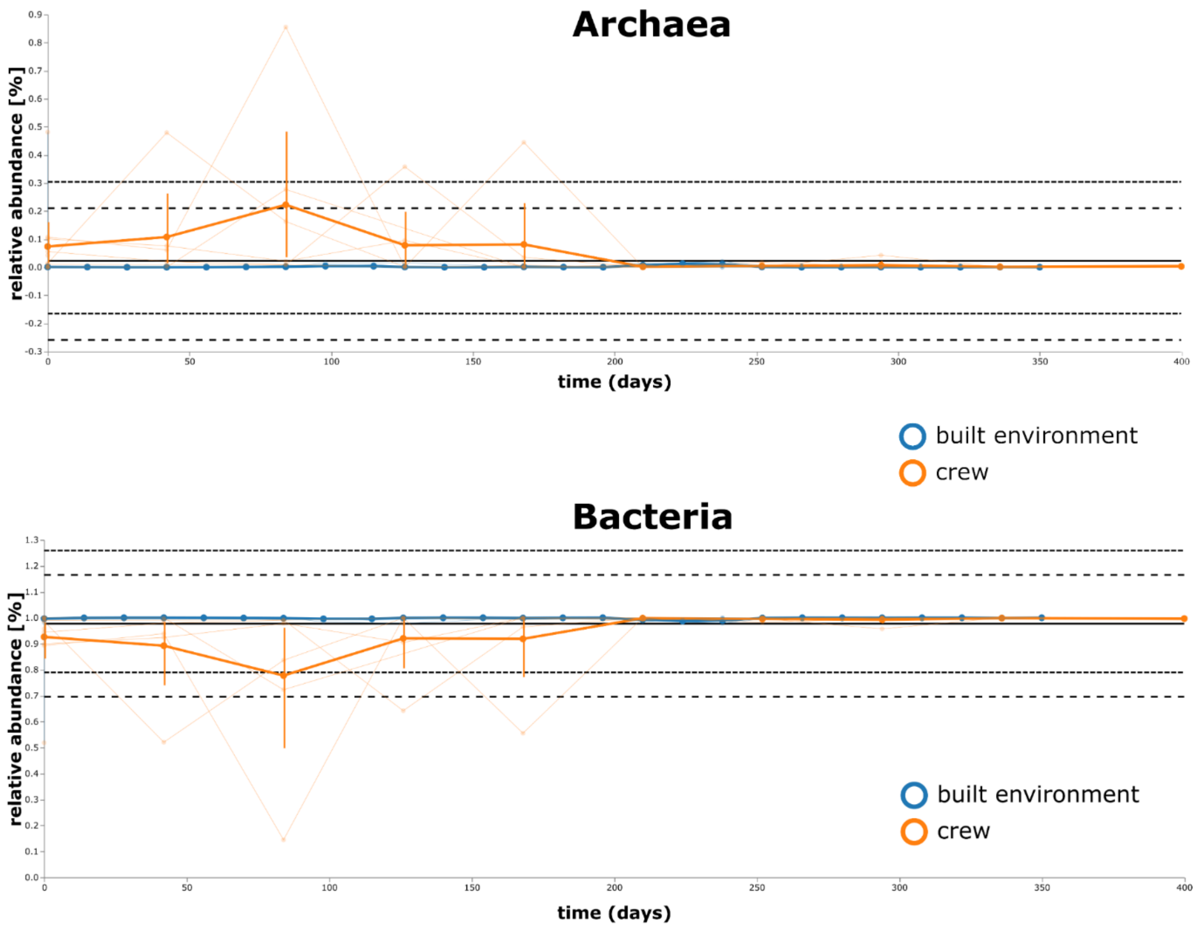

Supplementary Figure S22: Volatility analysis based on linear regression models with time of Archaea and Bacteria from the crew and the HI-SEAS habitat.

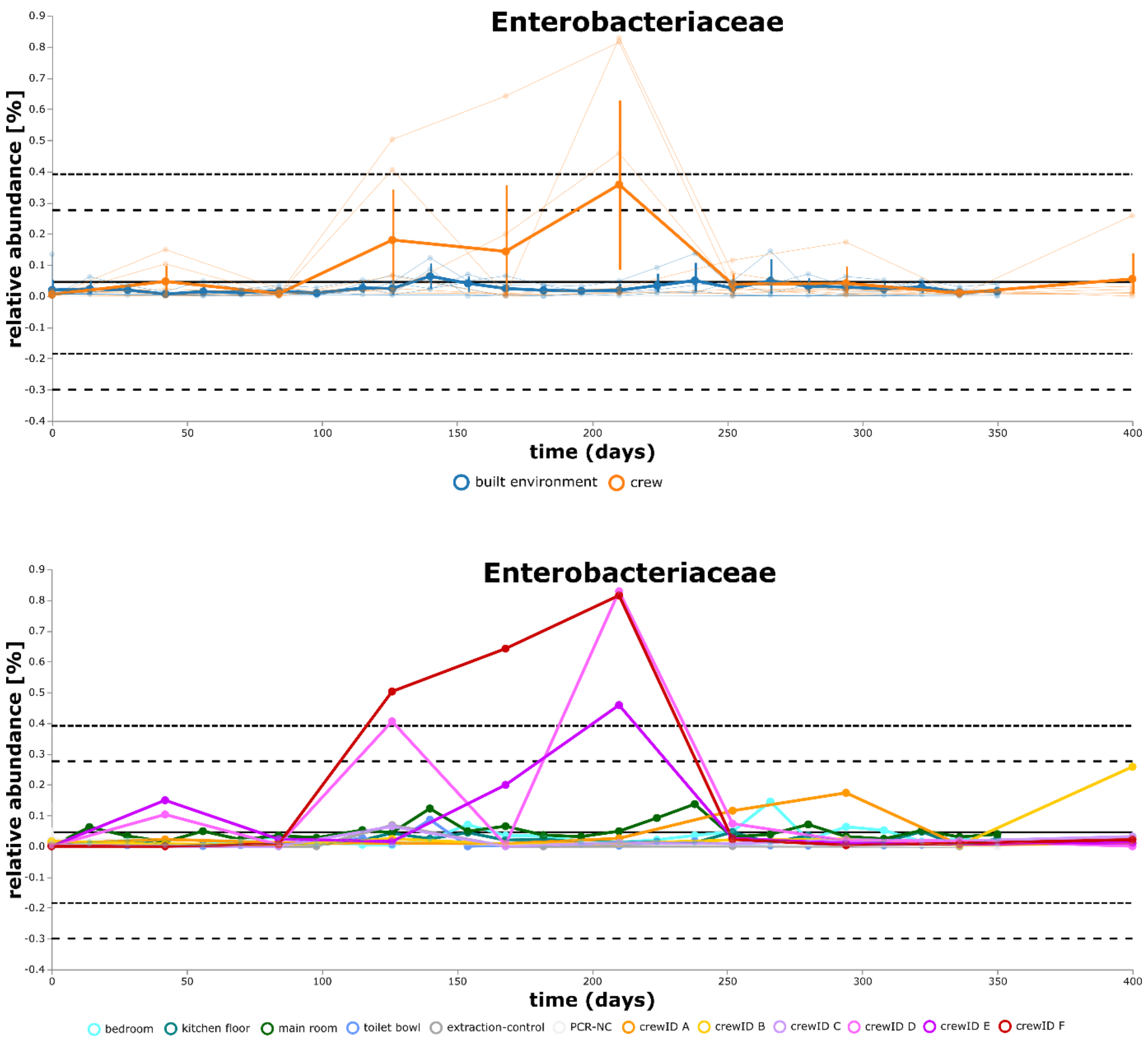

Supplementary Figure S23: Volatility analysis based on linear regression models with time of Enterobacteriaceae resolved to different sampling environments (built environment and crew) as well as individual crew members and sampling locations within the HI-SEAS habitat.

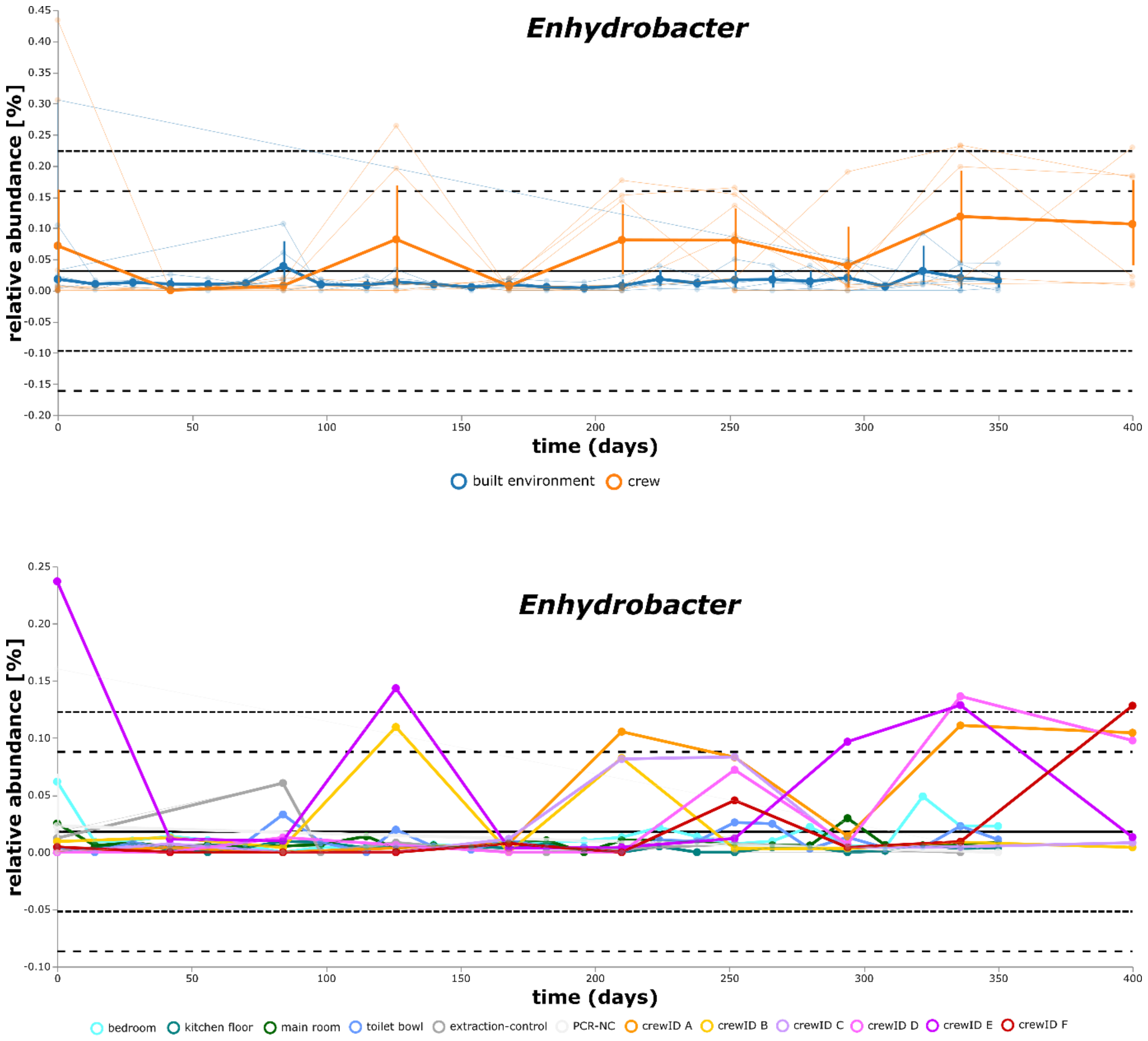

Supplementary Figure S24: Volatility analysis based on linear regression models with time of *Enhydrobacter* resolved to different sampling environments (built environment and crew) as well as individual crew members and sampling locations within the HI-SEAS habitat.

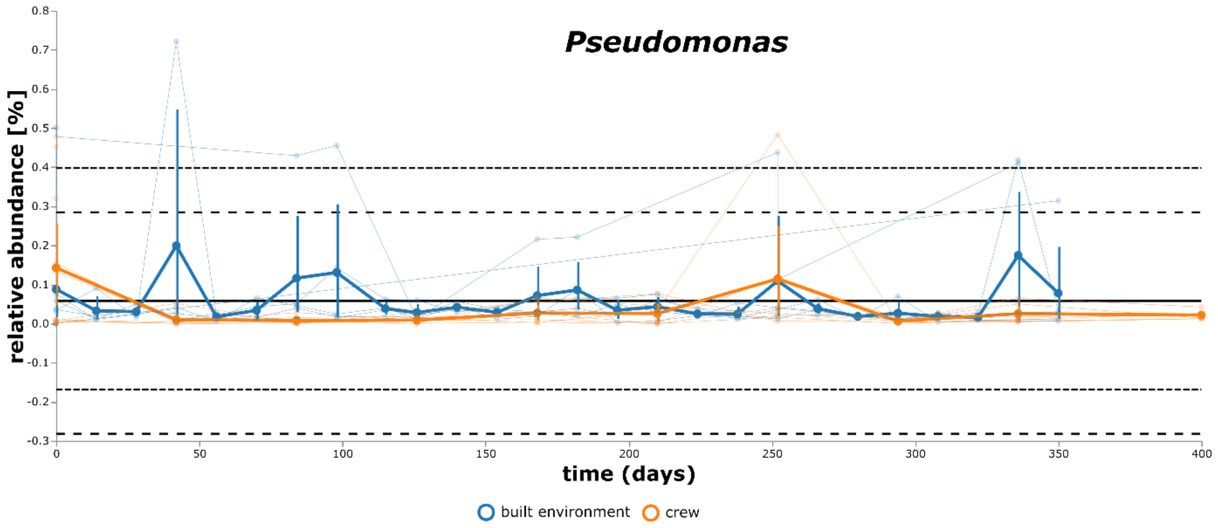

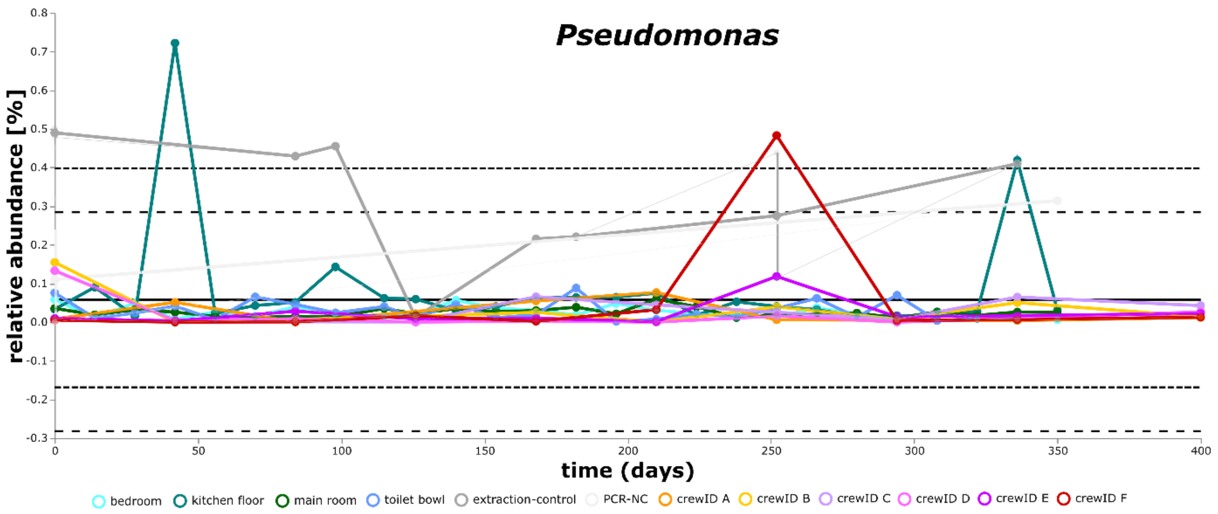

Supplementary Figure S25: Volatility analysis based on linear regression models with time of *Pseudomonas* resolved to different sampling environments (built environment and crew) as well as individual crew members and sampling locations within the HI-SEAS habitat.

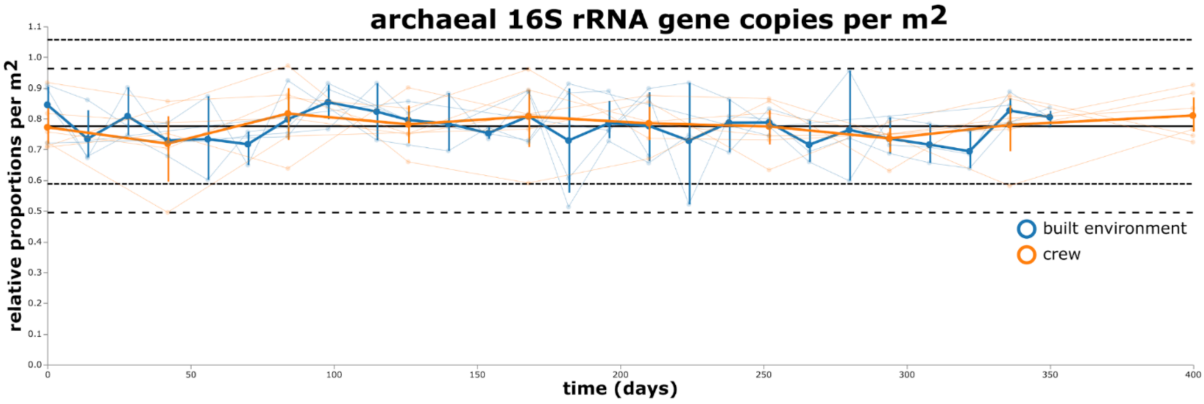

Supplementary Figure S26: Volatility analysis of archaeal 16S rRNA gene copies per m^2 according to qPCR.
